# Reliable reference genes for normalization of gene expression data in tea plants (*Camellia sinensis*) exposed to metal stresses

**DOI:** 10.1371/journal.pone.0175863

**Published:** 2017-04-28

**Authors:** Ming-Le Wang, Qing-Hui Li, Hua-Hong Xin, Xuan Chen, Xu-Jun Zhu, Xing-Hui Li

**Affiliations:** Tea Research Institute, College of Horticulture, Nanjing Agricultural University, Nanjing, Jiangsu, People’s Republic of China; Kumamoto University, JAPAN

## Abstract

Tea plants [*Camellia sinensis* (L.) O. Kuntze] are an important leaf-type crop that are widely used for the production of non-alcoholic beverages in the world. Exposure to excessive amounts of heavy metals adversely affects the quality and yield of tea leaves. To analyze the molecular responses of tea plants to heavy metals, a reliable quantification of gene expression is important and of major importance herein is the normalization of the measured expression levels for the target genes. Ideally, stably expressed reference genes should be evaluated in all experimental systems. In this study, 12 candidate reference genes (i.e., *18S rRNA*, *Actin*, *CYP*, *EF-1α*, *eIF-4α*, *GAPDH*, *MON1*, *PP2AA3*, *TBP*, *TIP41*, *TUA*, and *UBC*) were cloned from tea plants, and the stability of their expression was examined systematically in 60 samples exposed to diverse heavy metals (i.e., manganese, aluminum, copper, iron, and zinc). Three Excel-based algorithms (geNorm, NormFinder, and BestKeeper) were used to evaluate the expression stability of these genes. *PP2AA3* and *18S rRNA* were the most stably expressed genes, even though their expression profiles exhibited some variability. Moreover, commonly used reference genes (i.e., *GAPDH* and *TBP*) were the least appropriate reference genes for most samples. To further validate the suitability of the analyzed reference genes, the expression level of a phytochelatin synthase gene (i.e., *CsPCS1*) was determined using the putative reference genes for data normalizations. Our results may be beneficial for future studies involving the quantification of relative gene expression levels in tea plants.

## Introduction

Quantification of gene expression levels is an important part of the systematic characterization of gene transcriptional mechanisms and regulatory networks. The quantitative real-time polymerase chain reaction (qRT-PCR) is one of the most commonly used methods to quantify target genes expression levels due to its practical simplicity, specificity, reproducibility, and highly sensitivity in detecting transcripts with low copy numbers [1, 2]. An accepted standard procedure to conduct and interpret qRT-PCR experiments was lacking prior to 2009, which is when Bustin et al. [3] proposed the MIQE guidelines. Selecting appropriate reference genes is crucial for the reliable quantification of gene expression data [4]. To evaluate the stability of candidate reference genes in various experimental conditions, statistical algorithms have been developed, including geNorm [5], NormFinder [6], and BestKeeper [7]. An ideal reference gene should be stably transcribed under diverse experimental conditions [8]. However, it is unreasonable to expect the expression of any gene to be completely stable in a living cell. Thus, it is necessary to identify suitable reference genes to normalize expression data prior to investigating target genes expression levels.

Tea plants [*Camellia sinensis* (L.) O. Kuntze] originating from the Yunnan–Guizhou Plateau in southwestern China are an important perennial evergreen woody crop of the family *Theaceae* [9, 10]. Being rich in biologically active metabolites, such as tea polyphenols, theanine, and polysaccharides, tea leaves have long been used as the raw materials for dietary supplements, health foods, cosmeceuticals, and especially the production of non-alcoholic caffeine-containing beverages in the world [11, 12]. As sessile organisms, tea plants are continuously exposed to various adverse environmental conditions, such as drought stress [13], heat stress [14], salinity stress [15], and especially heavy metal stresses, which considerably affect tea growth, production, and quality [16–21]. For example, high concentration of Mn decreased tea production [16, 17]. Zn-stress decreased net photosynthetic rate, transpiration rate, stomatal conductance, growth and relative water content of *Camellia sinensis* considerably [18, 19]. Moreover, Yadav and Mohanpuria [20] demonstrated that Cu and Al exposure induces oxidative stress in *C*. *sinensis*. Additionally, excessive iron can adversely affect the quality of tea [21]. Identifying reliable reference genes under different environmental conditions is imperative for analyzing immediate molecular responses in *C*. *sinensis* cells.

Several suitable *C*. *sinensis* reference genes have been investigated, with some inconsistency observed in their expression levels under different experimental conditions [22–24]. *CsPTB1*, *CsEF1*, *CsSAND1*, *CsCLATHRIN1*, and *CsUBC1* are the top five most stably expressed reference genes under six experimental conditions (i.e., diurnal expression in leaves, expression in different organs, expression in leaves/shoots exposed to different cold and short day treatment, expression in shoots treated with an auxin antagonist, and expression in shoots treated with lanolin) [22]. *TUA1* is the most suitable reference gene for analyses of damaged tissues [23]. Additionally, *CsTBP* and *CsTIP41* displayed the maximum stability in tea leaf development, while *CsTBP* is also the most stably expressed gene in response to hormones [24]. However, a systematic approach for selecting reference genes useful for analyzing gene expression levels in *C*. *sinensis* plants in response to metal stresses has not been developed. Hence, identifying suitable reference genes in *C*. *sinensis* plants exposed to increasing metal concentrations is necessary, which will provide new information relevant to future research on molecular mechanism studies in tea plants.

In this study, we selected 12 candidate reference genes (i.e., *18S rRNA*, *Actin*, *CYP*, *EF-1α*, *eIF-4α*, *GAPDH*, *MON1*, *PP2AA3*, *TBP*, *TIP41*, *TUA*, and *UBC*) that were confirmed to be stably expressed in earlier studies [25–28]. The sequences of the 12 candidate genes were obtained based on our previously generated *C*. *sinensis* transcriptome sequencing data [29]. Specific details regarding these reference genes are listed in Table 1. We used qRT-PCR to determine gene expression levels in tea leves exposed to increasing concentrations of metals (i.e., Mn, Al, Cu, Fe, and Zn). Three different algorithms (i.e., geNorm, NormFinder, and BestKeeper) were used to evaluate the expression stability of the candidate reference genes. Additionally, *C*. *sinensis* phytochelatin synthase (CsPCS1), which is important for detoxifying the effects of heavy metals and was found to be up-regulated at its transcript level in response to Cu and Al stresses [20], was used to validate the reliability of the selected reference genes in tea leaves. This study is the first to analyze potential reference genes for investigating tea plants under heavy metal stress conditions. Our data may be useful for developing more accurate and reliable protocols to analyze the expression of other tea plant genes in response to metal stresses.

**Table 1:** The characteristics of primers used for quantitative real-time PCR in *C*. *sinensis*.

Gene symbol	*Arabidopsis* locus description	*Arabidopsis* homolog locus	Primer sequences (5'-3') forward/reverse	Amplicon length (bp)	Melting T_m_ (°C)	Efficiency (%)	Correlation coefficient (R^2^)
18S rRNA	18S ribosomal RNA	ATMG01390	ACACCCTGGGAATTGGTTT/ GTATGCGCCAATAAGACCAC	106	83.5	102.7	0.998
Actin	Actin 7	AT5G09810	CAGACCGTATGAGCAAGGAA/ GCTTAGGGATGCGAGGATAG	122	82.0	101.0	0.999
CYP	Cyclophilin	AT3G56070	TTTGCGGATGAGAACTTCAA/ CCATCTCCTTCACCACACTG	181	82.5	100.3	0.996
EF-1α	Elongation factor-1α	AT1G07940	CAAGCGTGTCATCGAGAGAT/ ATACCACGTTCACGTTCAGC	108	81.5	105.8	0.997
eIF-4α	Eukaryotic translation initiation factor 4α-1	AT3G13920	TGAGAAGGTTATGCGAGCAC/ GCAACATGTCAAACACACGA	149	83.0	104.3	1.000
GAPDH	Glyceraldehyde-3-phosphate dehydrogenase	AT1G42970	GACTGGAGAGGTGGAAGAGC/ AGCCATTCCAGTCAATTTCC	114	82.5	99.8	0.996
MON1	MONENSIN SENSITIVITY1	AT2G28390	ATTTCCTTCGTGGAGAATGG/ GCCCATAAACAAGCTCCAAT	160	82.0	98.9	0.999
PP2AA3	Protein phosphatase 2A subunit A3	AT1G13320	CAACATGTTCGCTCTGCTTT/ GGGAAAGGAATATTGGCAGA	100	81.0	101.6	0.995
TBP	TATA-box binding protein	AT1G55520	AAGGGATCCAAAGACGACAG/ TGAAATCCTTGAATTTGGCA	149	81.5	98.2	0.997
TIP41	TIP41-like family protein	AT4G34270	CGAAAGAGCCCATTCTCTTC/ ACGTGTGTCCCTCAATCTCA	173	80.5	104.5	0.997
TUA	Tubulin alpha-3	AT5G19770	TTTGGAGCGCTTGTCTGTAG/ TGTGTTCAAGGAGGGAATGA	134	82.0	102.3	0.996
UBC	Ubiquitin-protein ligase	AT4G27960	GACATGTTTCATTGGCAAGC/ ACCTTAGGTGGCTTGAATGG	116	81.0	101.7	0.995
CsPCS1	Phytochelatin synthase	AT5G44070	AATGCCCTTGCTATTGATCC/ CTCCAGAACAGTGAGCCAAA	151	81.0	103.4	0.992

## Materials and methods

### Plant materials and treatments

Two-year-old tea plants (*C*. *sinensis* cv. ‘Longjing-changyecha’) were collected from the fields of Gaochun District, Jiangsu Province, China (longitude: 118.57E, latitude: 31.19N). The plants were pre-incubated in a control nutrient solution [30] for 28 days (from September 8, 2015 to October 6, 2015) in a climate-controlled chamber under a 14-h light (24°C)/10-h dark (20°C) photoperiod (light intensity: 220 μmol m^–2^ s^–1^) and relative humidity of 75%. We then added MnSO_4_, Al_2_(SO_4_)_3_, CuSO_4_, FeSO_4_, or ZnSO_4_ for final concentrations of 50 μM, 12.5 mM, 13 μM, 210 μM, and 51 μM, respectively. After incubating the treated and control plants for 0, 1, 4, and 7 days, the fully expanded third leaves from the top bud of tea plants were harvested (Figure A in S1 File), immediately frozen in liquid nitrogen, and stored at −80°C.

### Total RNA extraction and cDNA synthesis

Total RNA was extracted using the *EasyPure*^®^ Plant RNA Kit (TransGen, Beijing, China). The concentration and purity of RNA samples were measured using the ONE Drop OD-1000+ spectrophotometer (ONE Drop, Shanghai, China). Only samples with an A_260_/A_280_ ratio of 1.8–2.0 and an A_260_/A_230_ ratio > 2.0 were used for the subsequent cDNA synthesis. The integrity of the purified RNA was further confirmed by 1.2% agarose gel electrophoresis. We generated cDNA from 1 μg total RNA using the *TransScript*^®^ All-in-One First-Strand cDNA Synthesis SuperMix for qPCR (One-Step gDNA Removal) (TransGen). The resulting cDNA was diluted 20-fold in distilled deionized water and analyzed in a qRT-PCR assay.

### Selection of candidate reference genes and primer design

We selected 12 candidate genes (i.e., *18S rRNA*, *Actin*, *CYP*, *EF-1α*, *eIF-4α*, *GAPDH*, *MON1*, *PP2AA3*, *TBP*, *TIP41*, *TUA*, and *UBC*) that were previously determined to be appropriate reference genes for qRT-PCR assays from the TAIR database (http://www.arabidopsis.org). Potential homologues of these genes were identified using our *C*. *sinensis* transcriptome sequencing data [29]. The qRT-PCR primers were designed with Primer PREMIER 5.0 software (*PREMIER* Biosoft International, Palo Alto, CA, USA) according to the Minimum Information for Publication of Quantitative Real-Time PCR Experiments (MIQE) guidelines [3]. The target sequences of the 12 *C*. *sinensis* candidate reference genes were cloned using Taq DNA polymerase (TaKaRa, Dalian, China). The PCR amplification was conducted in a 20 μl sample consisting of 10.9 μl double-distilled H_2_O, 2 μl 10×PCR Buffer, 1.6 μl dNTPs (2.5 mM each), 1.2 μl MgCl_2_ (25 mM), 2 μl template cDNA, 1 μl each primer (10 μM), and 0.3 μl of Taq DNA polymerase. The PCR conditions were as follows: 4 min at 95°C for denaturation; 35 cycles of 30 s at 95°C (denaturation), 30 s at 55°C (annealing), and 30 s at 72°C (extension); and a final step of 5 min at 72°C for extension. The resulting amplicons were purified and cloned into the pEASY-T1 Simple Cloning Vector (TransGen) for sequencing (Genscript, Nanjing, China). Primer sequences and amplicon characteristics are listed in Table 1.

### Quantitative real-time PCR assay

We conducted the qRT-PCR assay using the *TransStart*^®^ Tip Green qPCR SuperMix (TransGen) and a CFX96 Real-Time System (Bio-Rad, Hercules, CA, USA). The PCR solution (20 μl) contained 10 μl 2×*TransStart*^®^ Tip Green qPCR SuperMix, 0.2 μM each primer, 1 μl diluted cDNA, and nuclease-free water. The PCR reaction conditions were as follows: 95°C for 2 min; 40 cycles of 95°C for 10 s, 60°C for 15 s, and 72°C for 20 s; 72°C for 3 min. The final dissociation curve was obtained between 65°C and 95°C to verify primer specificity. Each assay included three technical and biological replicates, and involved a standard curve with six serial dilution points. Amplification efficiencies (E) were calculated using standard curves with satisfactory linear relationships (R^2^ > 0.99). The following equation was used to calculate the E-value (%): E = (10^[−1/slope]^ −1) × 100.

### Data analysis

The expression levels of the 12 genes were determined according to the quantification cycle (Cq) value. The raw data are listed in Table A in S1 File. Three different Microsoft Excel-based programs (i.e., geNorm v3.5 [5], NormFinder v0.953 [6], and BestKeeper v1.0 [7]) were used to determine the expression stability of the candidate genes. The raw data were analyzed using BestKeeper. However, for geNorm and NormFinder, the raw Cq values were converted into relative quantity values using the formula 2^−ΔCq^ (ΔCq = each corresponding Cq value–minimum Cq value; Table B in S1 File). Additionally, the fold change in expression of the target gene (*CsPCS1*; GenBank: KY264048) relative to the different reference genes at various time points was analyzed using the 2^–ΔΔCq^ method, in which ΔΔCq = (C_q, Target gene_−C_q, Reference gene_)_Day x_−(C_q, Target gene_−C_q, Reference gene_)_Day 0_ [31].

## Results

### Assessment of primer specificity and amplification efficiency

**Fig 1:**
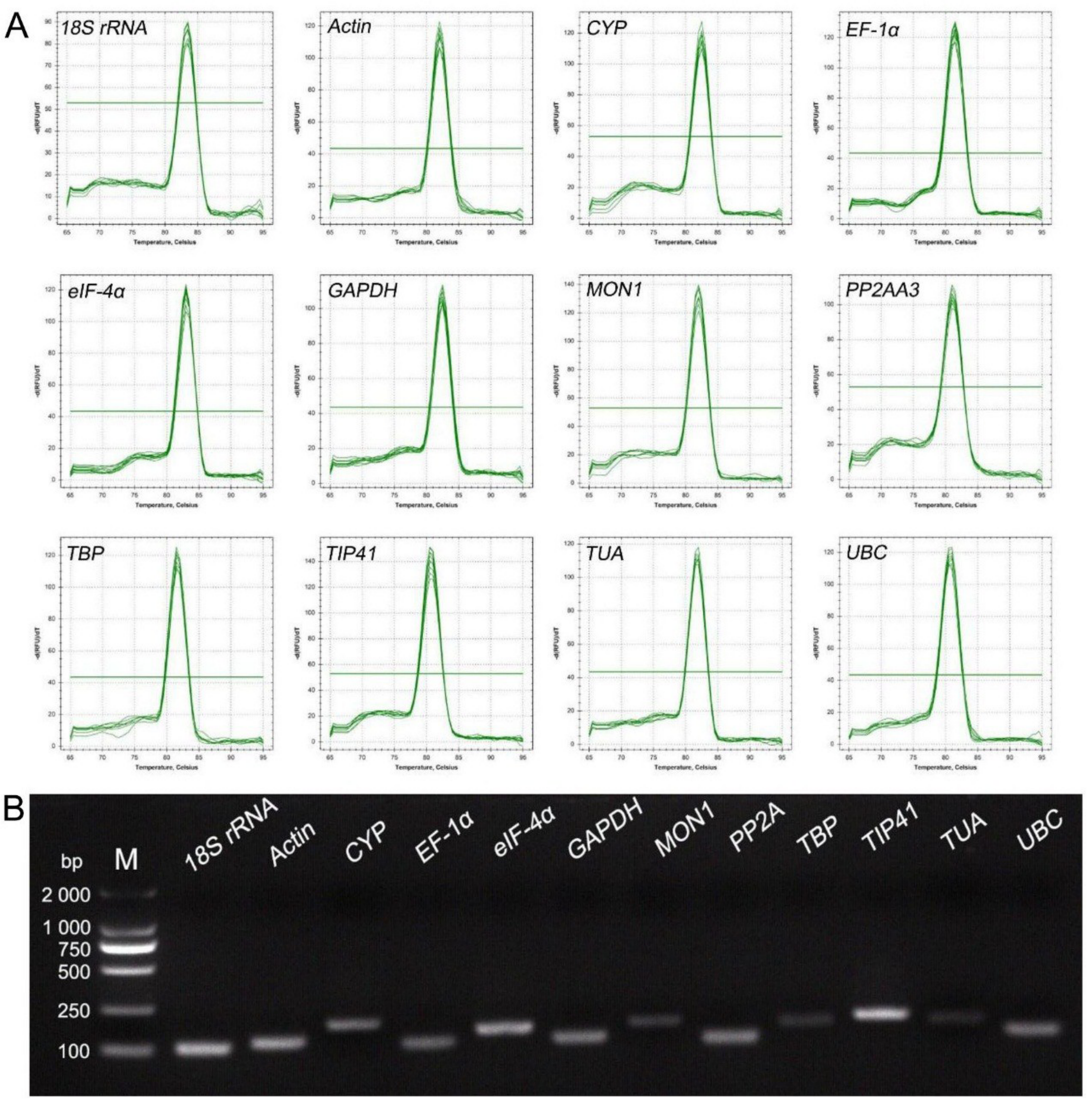
Confirmation of primer specificity and amplicon size. (a) Melting curve analysis of 12 candidate reference genes. (b) Amplification results for 12 candidate genes using a *C*. *sinensis* cDNA template. M: DL2000 DNA Marker.

We designed gene-specific primer pairs based on the sequences of the 12 candidate reference genes cloned from *C*. *sinensis* transcriptome sequences (Figures B–M and Table C in S1 File). Details regarding the 12 genes, primer sequences, amplicon lengths, and melting temperatures are privided in Table 1. The melting curve analysis revealed that all reactions produced a single distinct peak (Fig 1A). All primers amplified with a single PCR product of the expected size according to 1.5% agarose gel electrophoresis (Fig 1B). The estimated PCR amplification efficiencies for the candidate reference genes varied from 98.2% for *TBP* to 105.8% for *EF-1α*, and the correlation coefficients (R^2^) ranged from 0.995 for *UBC* to 1.000 for *eIF-4α* (Table 1).

### Quantification cycle values of candidate reference genes

**Fig 2:**
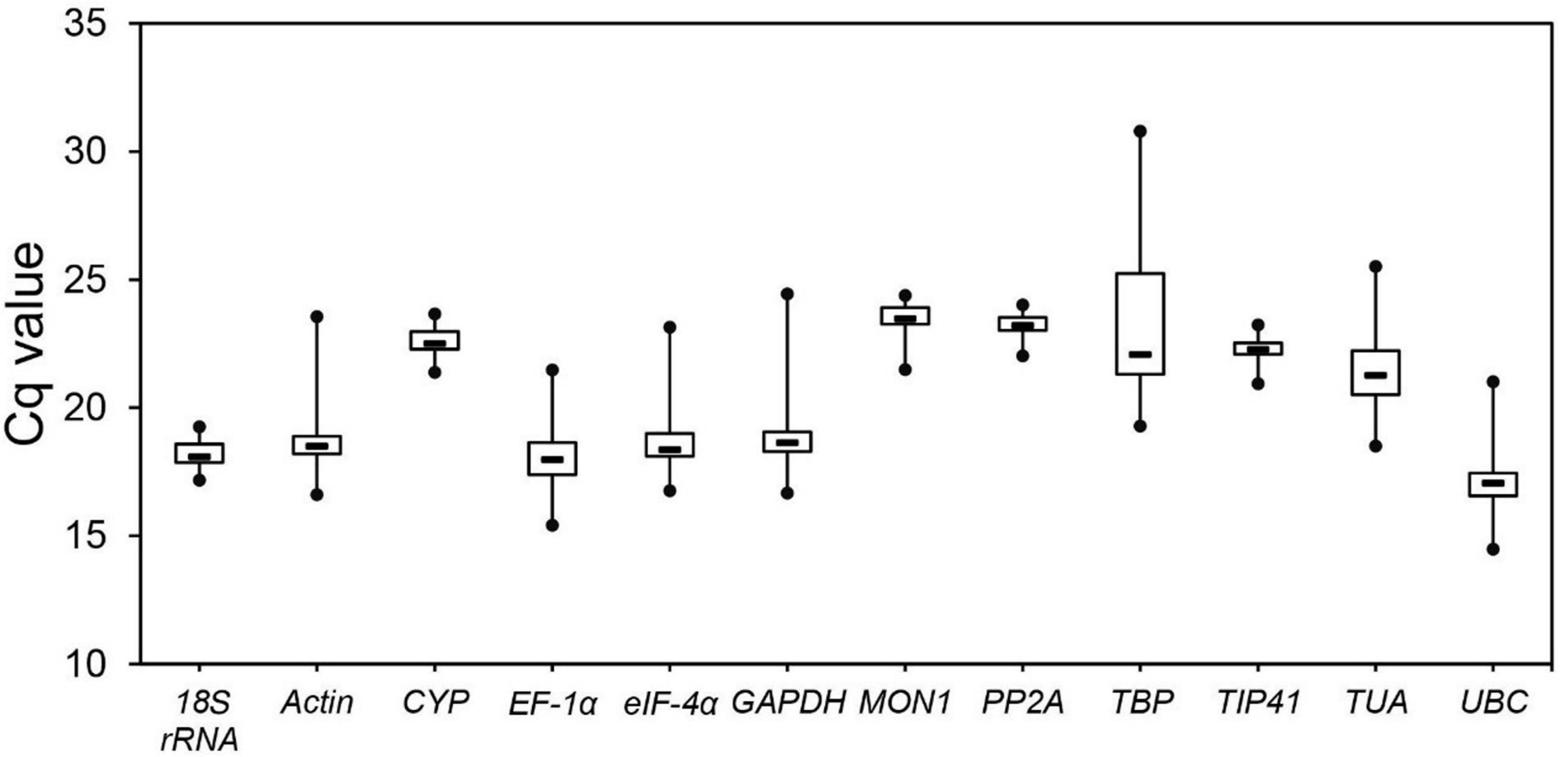
Quantification cycle (Cq) values of the 12 candidate reference genes in *C*. *sinensis* leaves under metal stresses. The lines across boxes represent the mean Cq values. The boxes indicate the 25th and 75th percentiles, while the whiskers correspond to the maximum and minimum values.

The Cq values in qPCR provided an overview of the gene expression levels of 12 candidate reference genes in 60 samples. There were apparent differences in the transcript abundance among genes. The raw data are listed in Table A in S1 File. The mean Cq values of all reference genes ranged from 17.08 to 23.51 (Fig 2). Low Cq value indicates the high expression levels, conversely mean the low expression levels. Among the 12 analyzed genes, *UBC* exhibited the highest expression levels with a mean Cq of 17.08, while *TBP* had the lowest expression levels with a mean Cq of 23.51 (Fig 2).

### Expression stability of candidate reference genes

**Fig 3:**
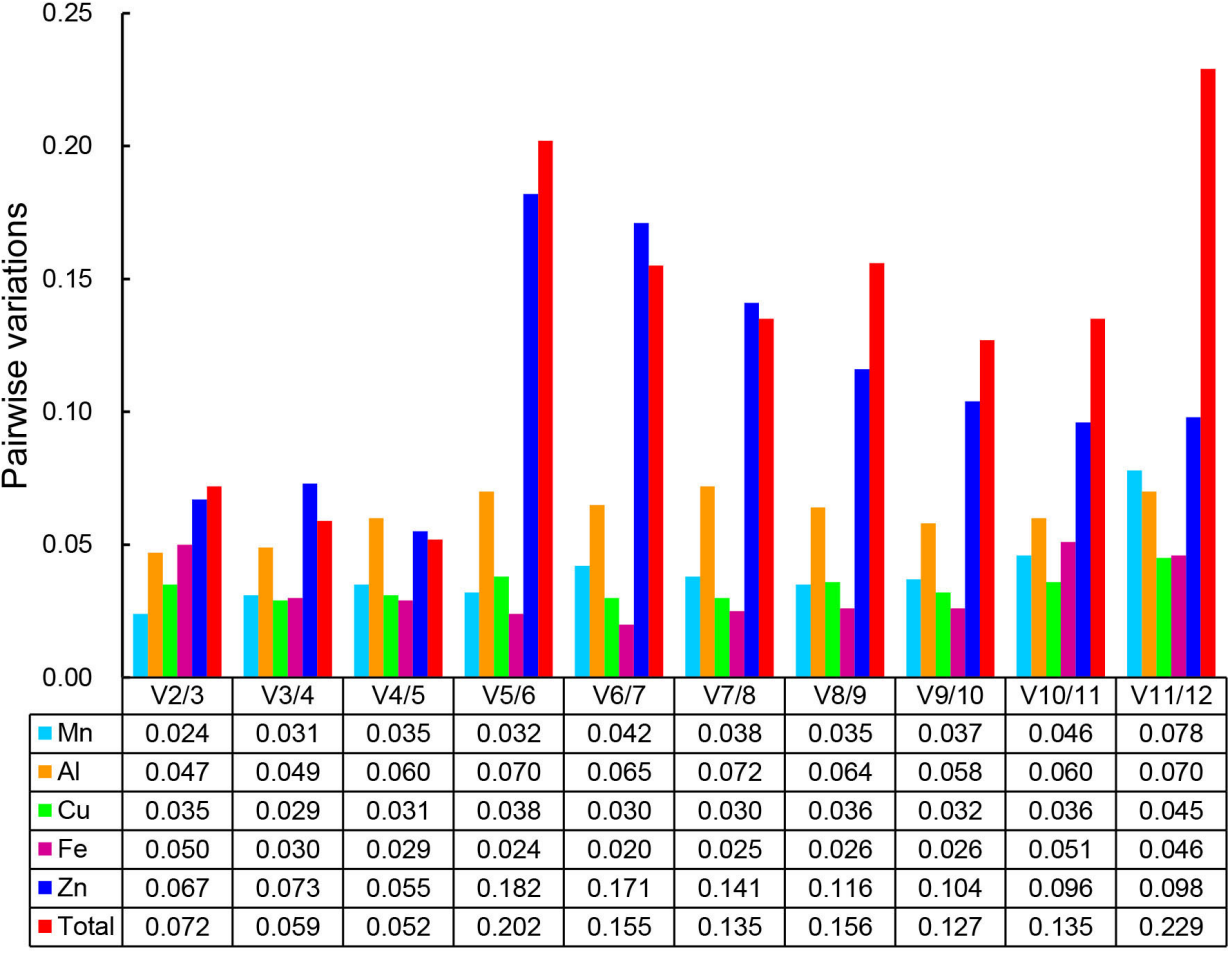
Determination of the optimal number of reference genes required for effective data normalization.

To identify the most suitable reference gene, three Microsoft Excel-based algorithms (geNorm, NormFinder, and BestKeeper) were used to analyze the stability of each reference gene. The geNorm analysis indicated that all genes performed well under individual stress conditions, with M values lower than the default limit of 1.5. However, the most suitable reference gene differed among treatments (Table 2). *PP2AA3* and *TBP* with same M values were the two best reference genes for Mn-treated leaves (Figure N in S1 File). Additionally, the most appropriate reference genes were *MON1* and *TIP41* in Al-treated leaves, *MON1* and *PP2AA3* in Cu-treated leaves, *EF-1α* and *eIF-4α* in Fe-treated leaves, and *CYP* and *PP2AA3* in Zn-treated leaves. *TUA* exhibited unstable expression in all samples. The pairwise variation [i.e., Vn/n+1 (n ≥ 2)] of the geNorm program was also used to determine the optimal number of reference genes required for data normalizations. Values (V2/3) lower than the recommended threshold of 0.15 indicated that two reference genes were sufficient for normalizing the gene expression data resulting from the exposure to the five metal stress conditions (Fig 3).

**Table 2:** Gene expression stability ranked by geNorm, NormFinder, and BestKeeper software programs. SD: standard deviation; CV: coefficient of variation.

Rank	geNorm		NormFinder		BestKeeper		
	Gene	Stability	Gene	Stability	Gene	SD	CV
1	*TIP41*	0.22	*Actin*	0.129	*PP2AA3*	0.37	1.58
2	*MON1*	0.22	*EF-1α*	0.134	*CYP*	0.39	1.71
3	*PP2AA3*	0.24	*TUA*	0.146	*MON1*	0.42	1.78
4	*18S rRNA*	0.25	*UBC*	0.164	*TIP41*	0.42	1.91
5	*CYP*	0.27	*18S rRNA*	0.169	*18S rRNA*	0.41	2.28
6	*UBC*	0.59	*TIP41*	0.172	*TUA*	1.08	5.05
7	*EF-1α*	0.75	*MON1*	0.173	*EF-1α*	0.91	5.07
8	*TUA*	0.86	*PP2AA3*	0.174	*UBC*	0.87	5.09
9	*Actin*	0.99	*GAPDH*	0.181	*Actin*	1.03	5.47
10	*eIF-4α*	1.08	*eIF-4α*	0.189	*GAPDH*	1.25	6.51
11	*GAPDH*	1.16	*CYP*	0.193	*eIF-4α*	1.37	6.92
12	*TBP*	1.44	*TBP*	0.24	*TBP*	1.72	7.64

The results of the NormFinder analysis revealed that the genes with the lowest values were the most stably expressed (Table 2). The three most stably expressed references genes for all samples were *Actin* (0.129), *EF-1α* (0.134), and *TUA* (0.146). *Actin* was the most stably expressed gene in Mn- and Fe-treated tea leaves (Table D in S1 File). In contrast, *18S rRNA* was the most stably expressed gene in response to Al and Cu treatments. Moreover, *UBC* (0.138) was likely the most suitable reference gene in Zn-treated leaves.

Lower standard deviations and coefficients of variation during the BestKeeper analysis corresponded to more stable gene expression. According to the BestKeeper rankings, *PP2AA3* was the most stably expressed gene in Al- and Fe-treated leaves (Table E in S1 File). Additionally, *TIP41* and *MON1* were the most stably expressed genes in Mn- and Cu-treated leaves, respectively. *UBC* expression levels were the most unstable in Zn-treated leaves, which contradicted the NormFinder results that suggested *UBC* is a good reference gene.

### Validation of selected reference genes

**Fig 4:**
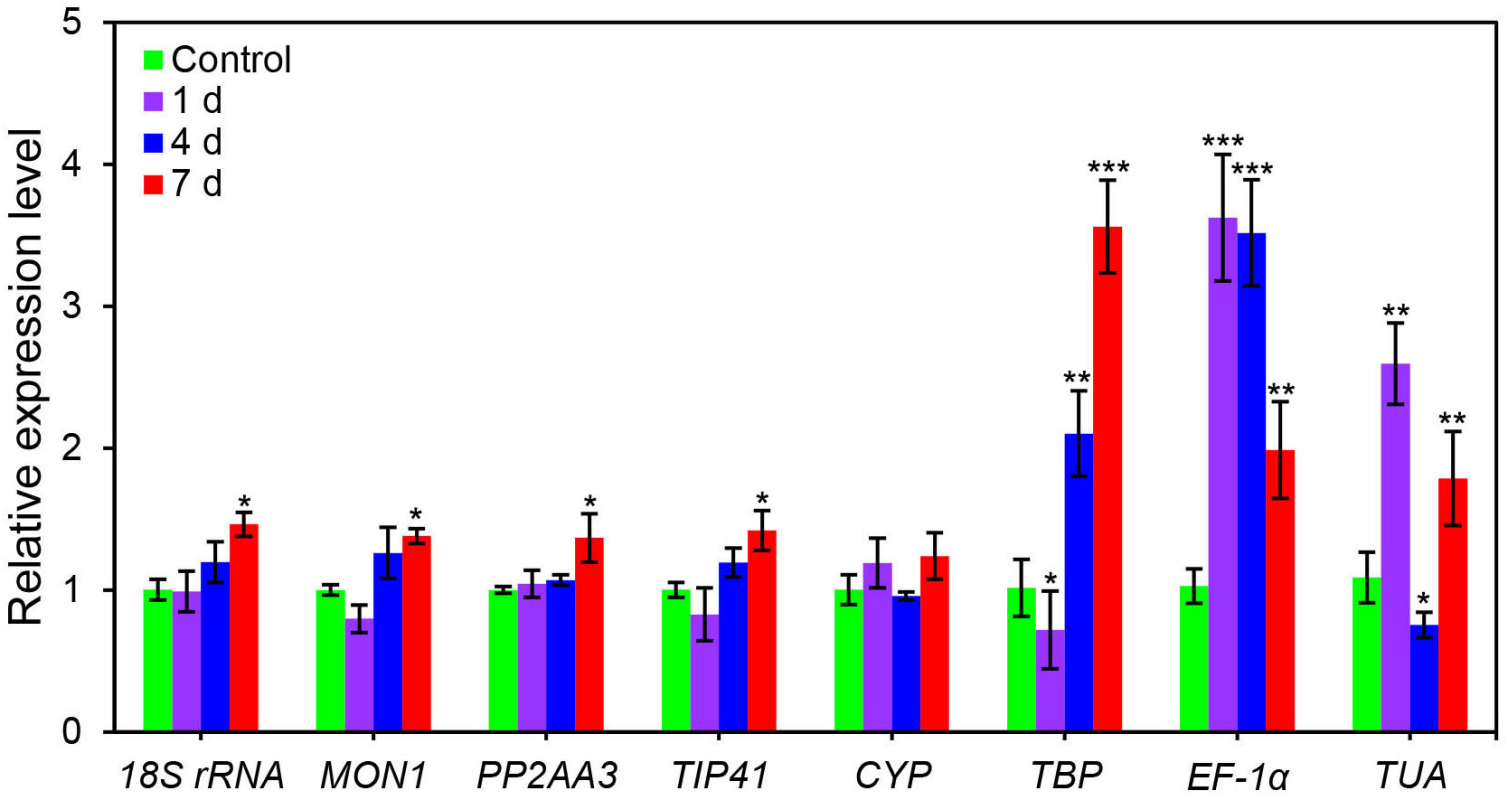
Relative quantification of *CsPCS1* gene expression using candidate reference genes in Al-stressed *C*. *sinensis* leaves. Data are presented as the means ± standard deviation of four replicates. Significant differences were determined by Duncan’s multiple range test (* *P* < 0.05, ** *P* < 0.01, and ** *P* < 0.001).

*CsPCS1* gene which is related to metal stresses was selected to further evaluate the reliability of the potential *C*. *sinensis* reference genes in qRT-PCR assays [20, 32]. The relative *CsPCS1* expression level following an Al treatments was determined using the expression levels of *18S rRNA*, *MON1*, *PP2AA3*, *TIP41*, *CYP*, *TBP*, *EF-1α*, and *TUA* to normalize data (Fig 4). Data normalizations using the more stably expressed reference genes (i.e., *18S rRNA* and *MON1*) resulted in consistent *CsPCS1* expression patterns, with the highest expression level observed following a 7-day exposure to Al. Similar expression patterns were observed when the less stably expressed reference genes were used to normalize data (i.e., *TIP41*, *CYP*, and *TBP*). However, when the least stably expressed genes were used for data normalization (i.e., *EF-1α* and *TUA*), the expression level of *CsPCS1* was considerably biased. This results indicated that the least stable genes *EF-1α* and *TUA* failed to standardize the expression data effectively.

## Discussion

In recent years, qRT-PCR has become an outstanding technique for studying gene expression profiles because of its accuracy, sensitivity, and reproducibility. To reach its maximum analytical potential, it is necessary to introduce appropriate internal reference genes or housekeeping genes for normalizing data. Sun et al. [33] were the first researchers to identify suitable *C*. *sinensis* reference genes for qRT-PCR analyses. They determined that *Csβ-actin* (GenBank: HQ420251) and *CsGAPDH* (GenBank: GE651107) could be used as reference gene during analyses of different tissues and leaf developmental stages, respectively. Afterwards, *Csβ-actin* and *CsGAPDH* have been widely applied for gene expression analyses in tea plants [14, 34, 35]. However, it is misleading to use previously identified reference genes for normalization without first investigating the stability of their expression levels under specific experimental conditions. Therefore, *18S rRNA*, *Actin*, *CYP*, *EF-1α*, *eIF-4α*, *GAPDH*, *MON1*, *PP2AA3*, *TBP*, *TIP41*, *TUA*, and *UBC* were selected to be validated under the experimental conditions of the present study, because they were observed to be stably expressed under various abiotic stresses, in particular in response to heavy metals [36–39].

Expression levels were determined as Cq values by qRT-PCR. The mean Cq values of the genes ranged from 17.08 (*UBC*)–23.51 (*TBP*), and the Cq values for all the tested samples were between 15 and 30. Here, the Cq values of *PP2AA3*, *18S rRNA*, and *CYP* (Cq_max_−Cq_min_ < 2 cycles; SD = 0.47, 0.50, and 0.46, respectively) were distributed more centrally than those of the other candidate genes, whereas the Cq value of *TBP* showed the highest variation (Cq_max_−Cq_min_ > 10 cycles, SD = 3.03). The Cq value comparison can provide a rough estimate on stability of gene expression, but not sufficient for accurate evaluation of expression patterns of reference genes [40, 41].

Three statistical algorithms (i.e., geNorm, NormFinder, and BestKeeper) were further used to determine which reference gene is best suited for transcript normalization in tea leaves exposed to heavy metals. Our geNorm results (V2/3 value < 0.15) revealed that two reference genes were sufficient for qRT-PCR data normalizations for the aforementioned five heavy metal stresses, indicating that the addition of third gene had no significant effect for normalization. In Mn-treated tea leaves, the geNorm analysis indicated that *PP2AA3*, *TBP*, and *UBC* were the most appropriate reference genes. Although *TBP* was identified as the best reference gene according to the geNorm results, it was only the seventh most suitable gene based on the BestKeeper analysis. NormFinder, geNorm, and BestKeeper ranked *Actin* as the best, third-best, and sixth-best reference gene, respectively. Additionally, *TIP41* was identified as the best reference gene by the BestKeeper program, while it was only the eighth-best and seventh-best reference gene according to geNorm and NormFinder, respectively. Based on these results, *PP2AA3* combined with *TBP* or *UBC* were recommended as the best combination of stable reference genes for normalizing gene expression levels in Mn-treated tea leaves. Similarly, *18S rRNA* combined with *PP2AA3* or *TIP41* are sufficient for analyses of Al-treated tea leaves. *MON1* combined with *18S rRNA* or *TIP41* should be used for Cu treatments, *eIF-4α* combined with *Actin* or *CYP* are suitable for Fe treatments. Finally, *PP2AA3* combined with *CYP* or *18S rRNA* are sufficient for analyzing Zn-treated tea leaves.

During previous analyses of gene expression levels in tea plant, *CsGAPDH* (GenBank: GE651107) was often used to normalize qRT-PCR data [13, 42, 43] because it was stably expressed in many other plant species [44–46]. However, according to geNorm and BestKeeper results of our study, *GAPDH* was identified as the least stably expressed reference gene in Zn- and Mn-treated tea leaves. These results highlight the fact that there is no single universal reference gene that is stably expressed under all experimental conditions [47–49] and that reference genes should be reconfirmed according to specific experimental conditions [50, 51].

To validate the suitability of potential reference genes, the expression profiles of *CsPCS1* was assessed in Al-treated tea leaves, with *18S rRNA*, *MON1*, *PP2AA3*, *TIP41*, *CYP*, *TBP*, *EF-1α*, and *TUA* as internal reference genes. The *CsPCS1* expression patterns were similar when the gene expression data were normalized using the most stably expressed reference gene (i.e., *18S rRNA*) and less stably expressed reference genes (i.e., *PP2AA3*, *MON1*, and *TIP41*). In contrast, when *TBP*, *EF-1α*, and *TUA* were used for data normalizations, the expression patterns and transcript levels were obviously different from those obtained following data normalizations with *18S rRNA* and other suitable reference genes. Hence, using a stable reference gene is a prerequisite for accurate relative quantifications of gene expression levels [52, 53].

## Conclusions

To the best of our knowledge, this is the first report describing the identification of suitable reference genes for qRT-PCR analyses in *C*. *sinensis* leaves exposed to heavy metal stress. The stability analysis of gene expression by geNorm, NormFinder, and BestKepper indicated that no single gene was stably expressed under different metal stress conditions. We have identified *PP2AA3*, *UBC*, and *TBP* as suitable reference genes under Mn stress. *18S rRNA*, *PP2AA3*, and *TIP41* were the most stably expressed genes under Al stress. *MON1*, *TIP41*, and *18S rRNA* were the most stable ones under Cu stress; *eIF-4α*, *CYP*, and *Actin* under Fe stress; and *PP2AA3*, *CYP*, and *18S rRNA* under Zn stress. Therefore, reference genes should be selected according to the specific metal stress being investigated. Moreover, the stability of previously used reference genes should be re-assessed to increase the accuracy of expression data and to avoid error propagation under certain experimental conditions. Additionally, the analysis of *CsPCS1* expression levels confirmed the importance of selecting appropriate reference genes for the normalization of qRT-PCR data. The reference genes selected in this study provide more choices for further gene expression analysis and functional studies in *C*. *sinensis*.

## Supporting information

S1 File Contains Figures. A-N and Tables A-E.
(DOCX)
