# Supplementary material for: Reliable reference genes for normalization of gene expression data in tea plants (Camellia sinensis) exposed to metal stresses
Source: PLoS One. 2017 Apr 28;12(4):e0175863. doi: 10.1371/journal.pone.0175863 (PMC5409199; doi:10.1371/journal.pone.0175863)
Supplement: S1 File — Contains Figures. A-N and Tables A-E. (DOCX) [file pone.0175863.s001.docx]

Supporting Information


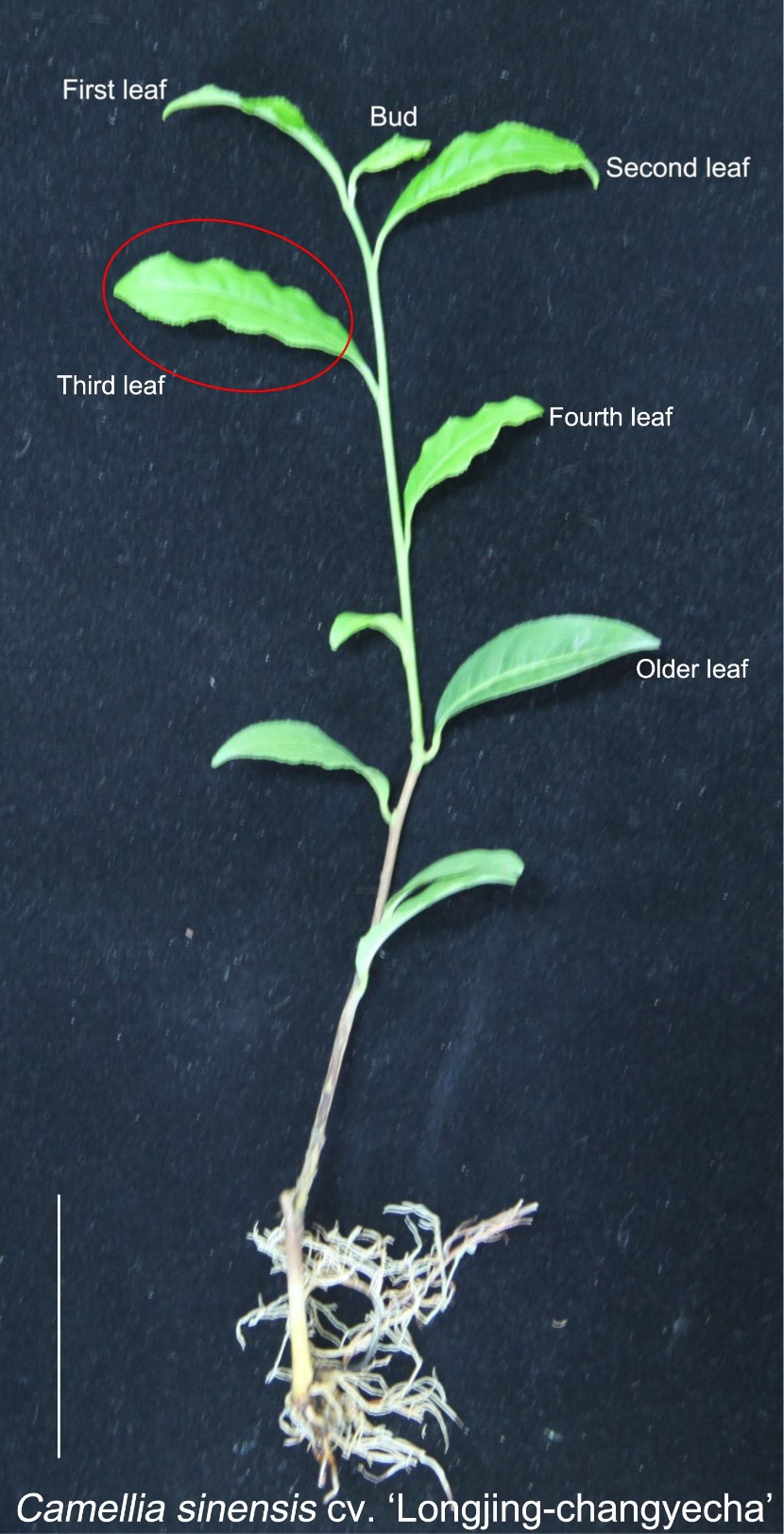


**Figure A. Photograph of tea plant (*Camellia sinensis* cv. ‘Longjing-changyecha’).** The third leaves under different metal-stressed leaves were collected for RNA extraction. Bar = 2 cm.


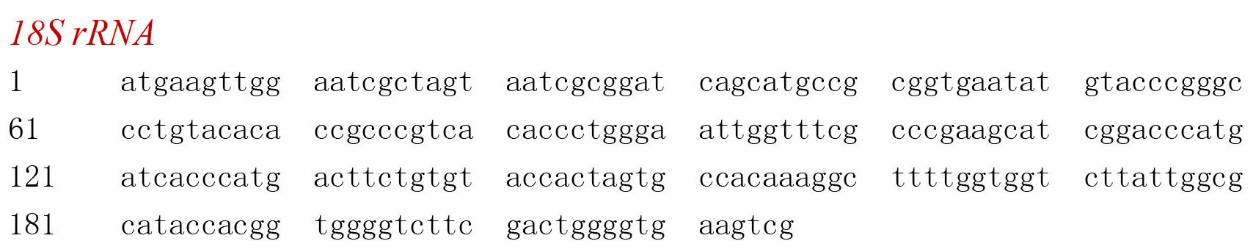


**Figure B. Nucleotide acid sequence of *18S rRNA* gene from *C. sinensis*.**


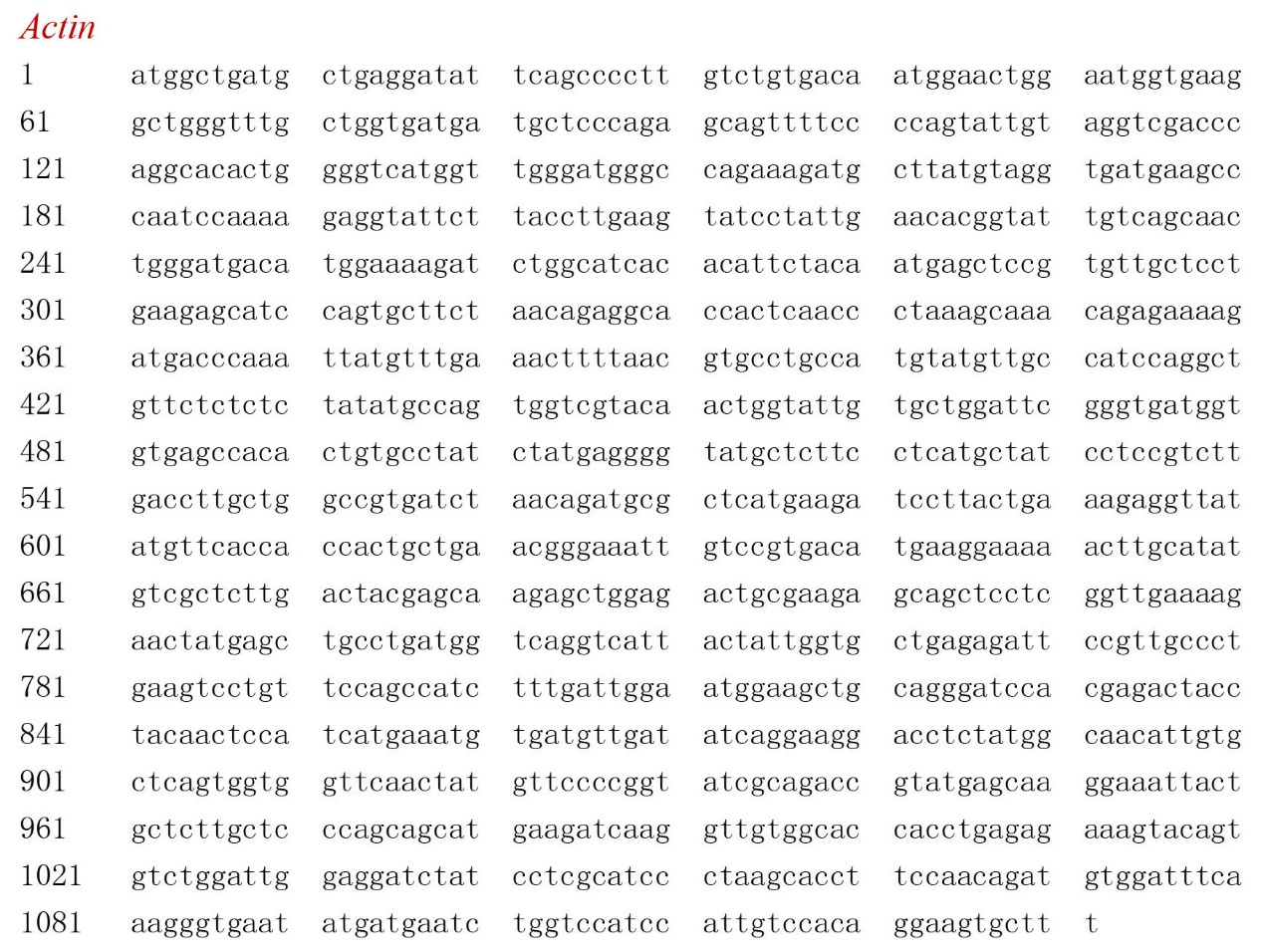


**Figure C. Nucleotide acid sequence of *Actin* gene from *C. sinensis*.**


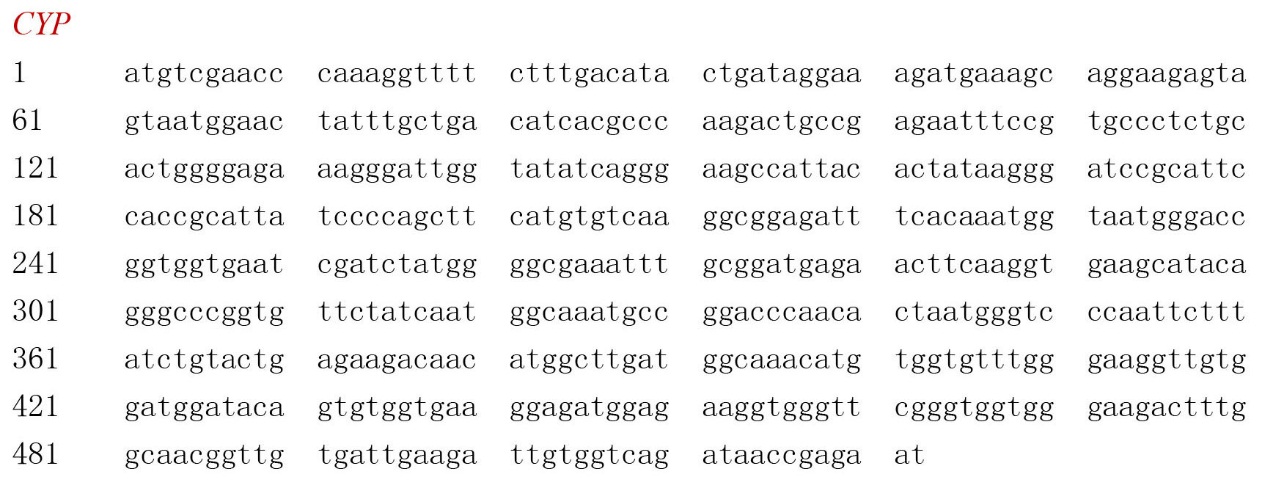


**Figure D. Nucleotide acid sequence of *CYP* gene from *C. sinensis*.**


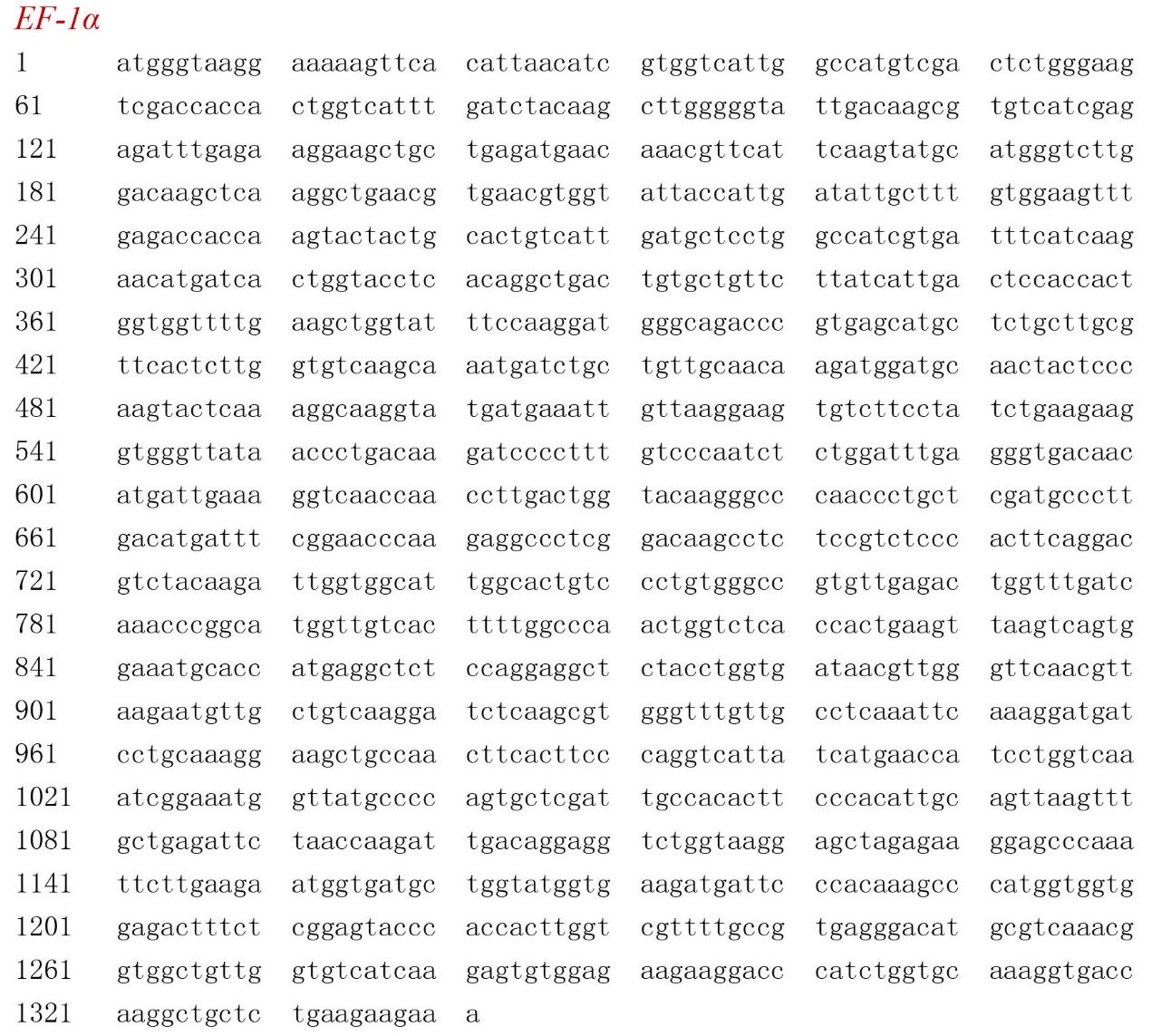


**Figure E. Nucleotide acid sequence of *EF-1α* gene from *C. sinensis*.**


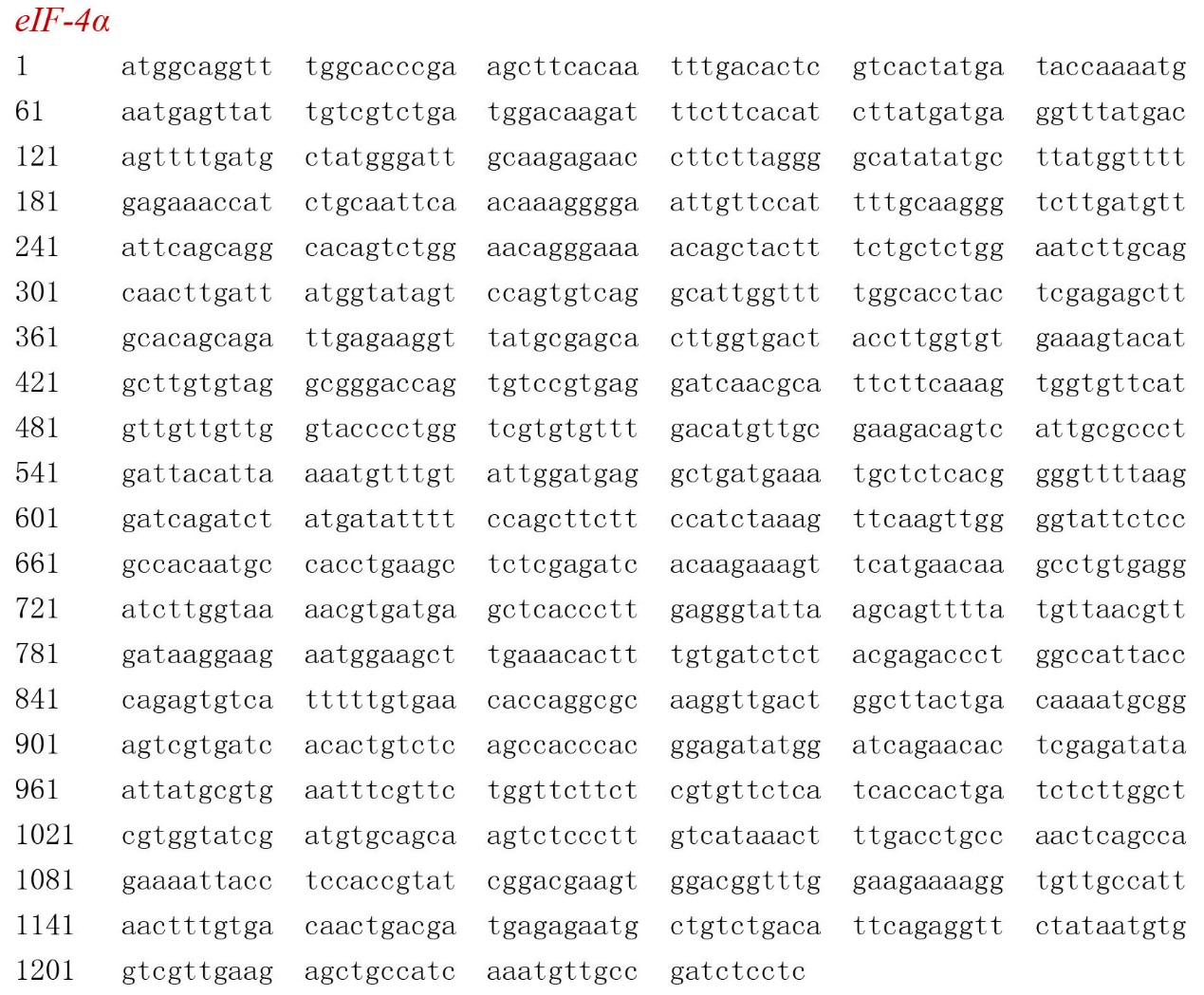


**Figure F. Nucleotide acid sequence of *eIF-4α* gene from *C. sinensis*.**


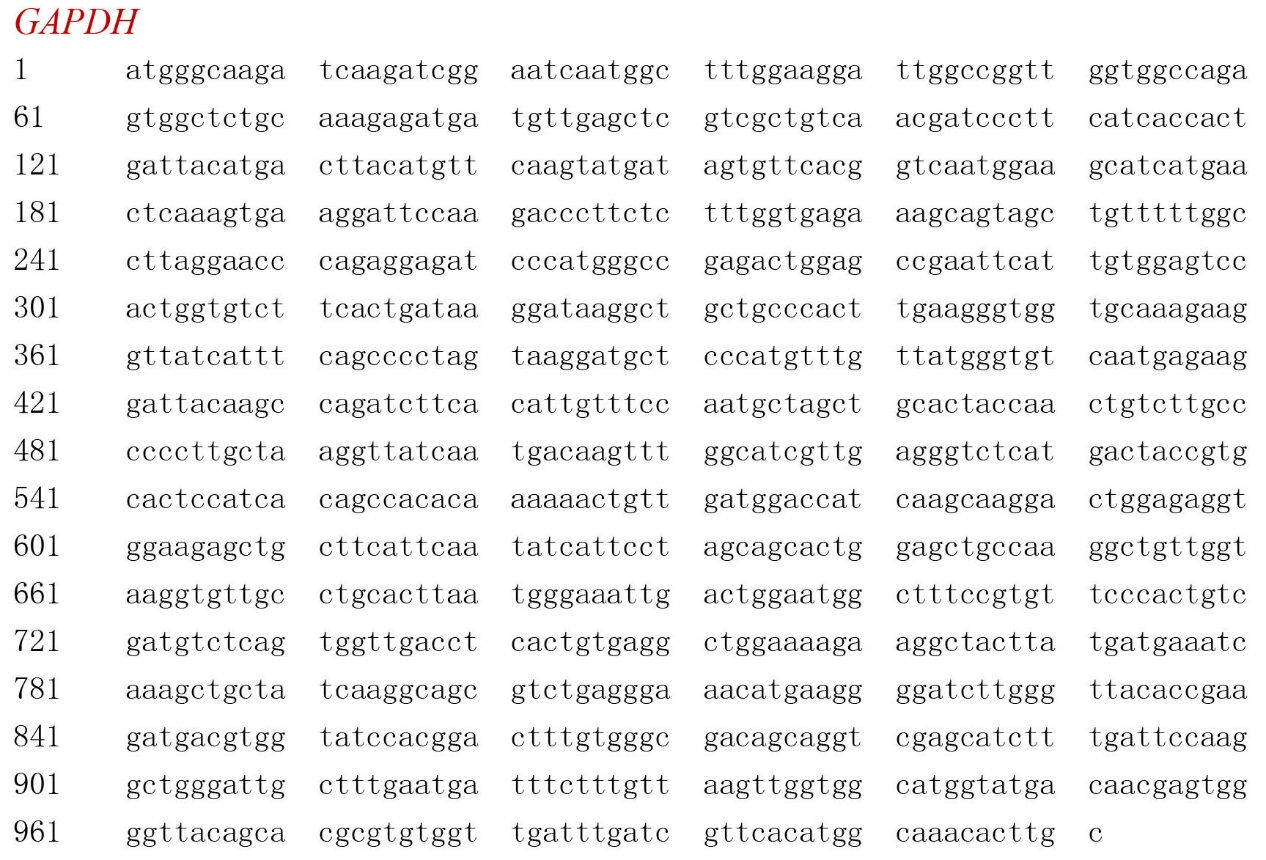


**Figure G. Nucleotide acid sequence of *GAPDH* gene from *C. sinensis*.**


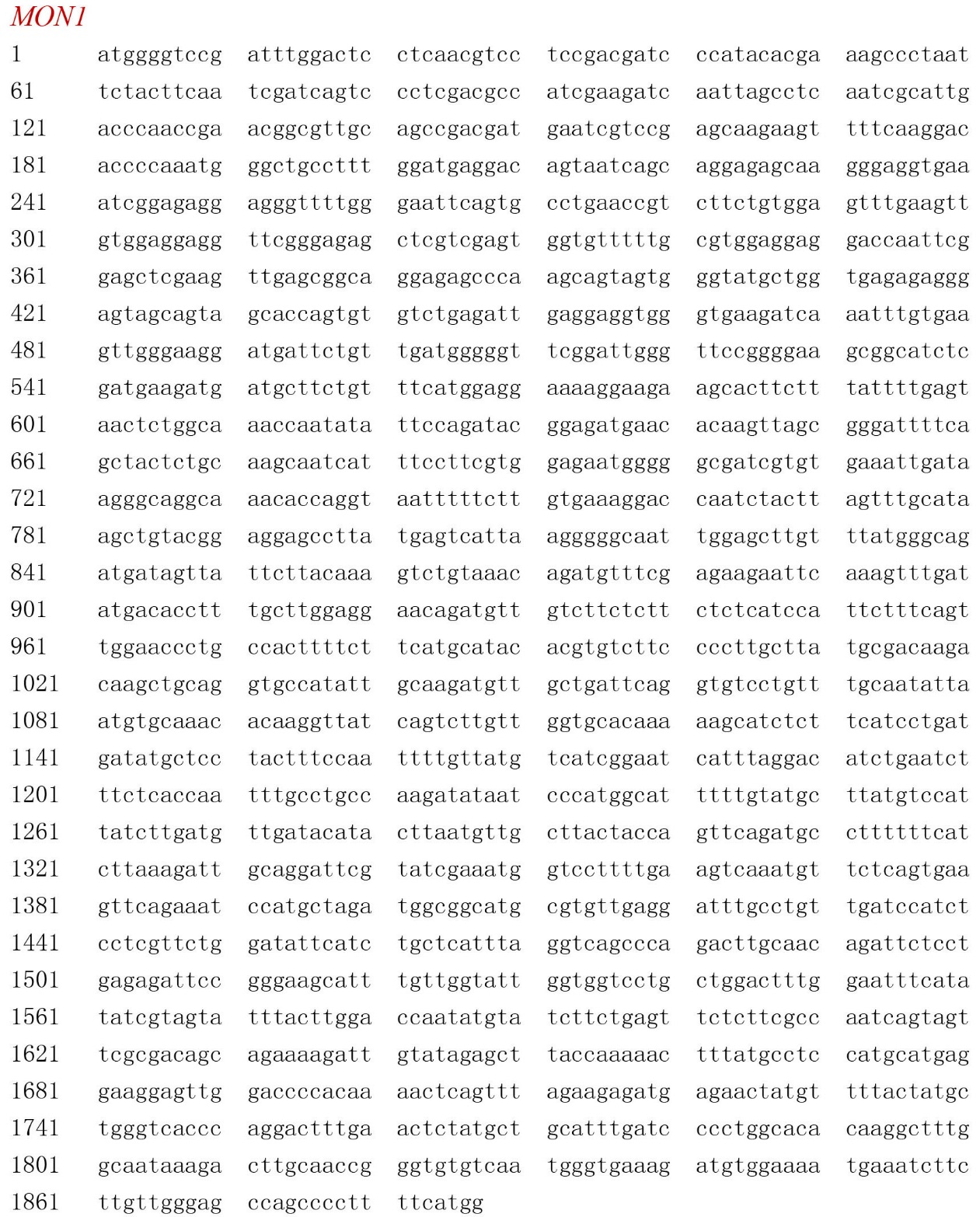


**Figure H. Nucleotide acid sequence of *MON1* gene from *C. sinensis*.**


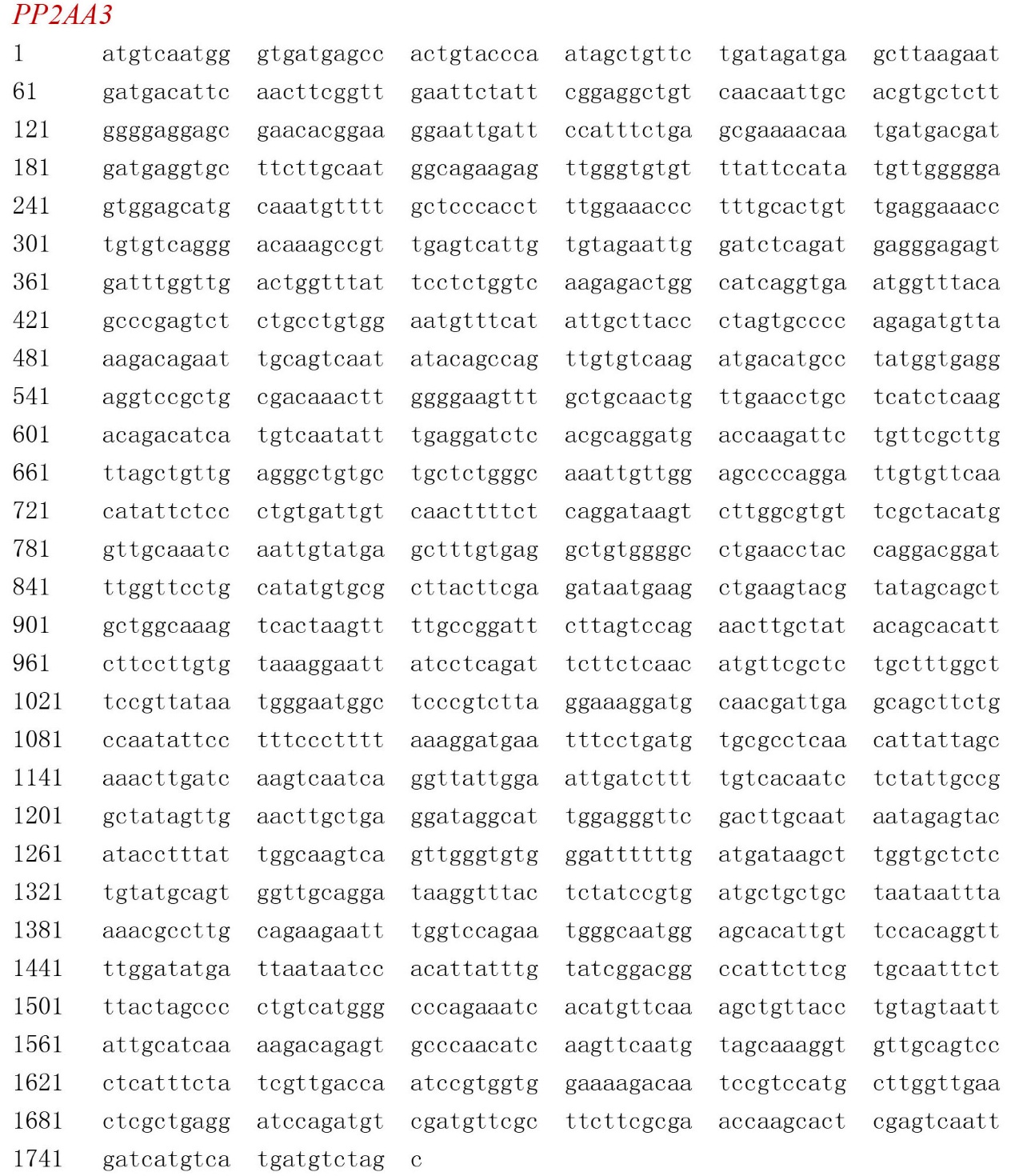


**Figure I. Nucleotide acid sequence of *PP2AA3* gene from *C. sinensis*.**


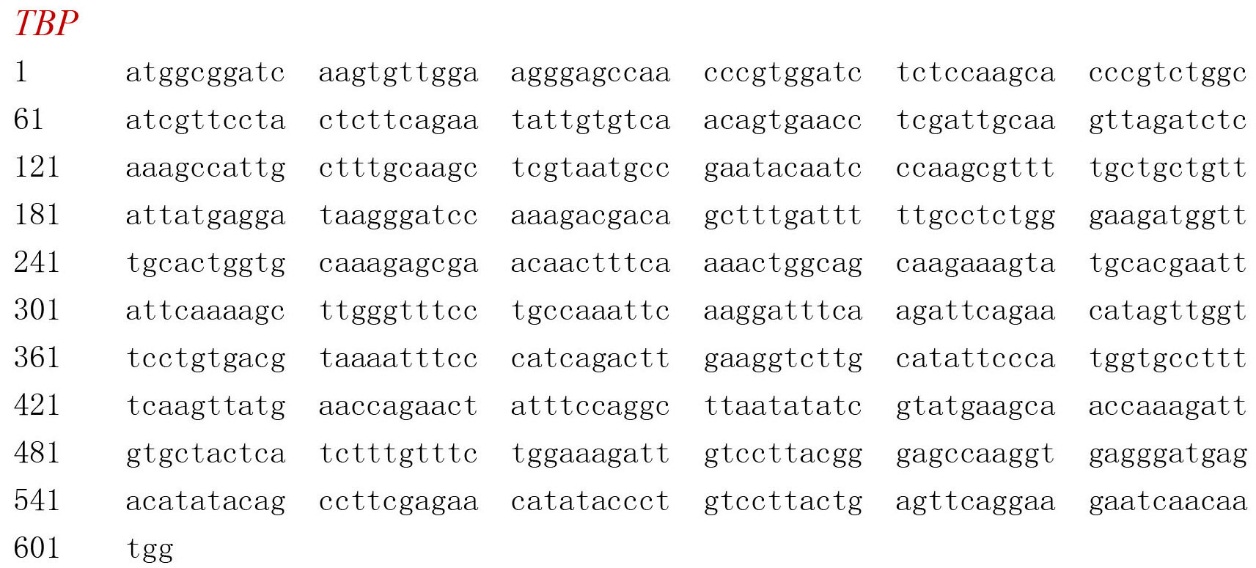


**Figure J. Nucleotide acid sequence of *TBP* gene from *C. sinensis*.**


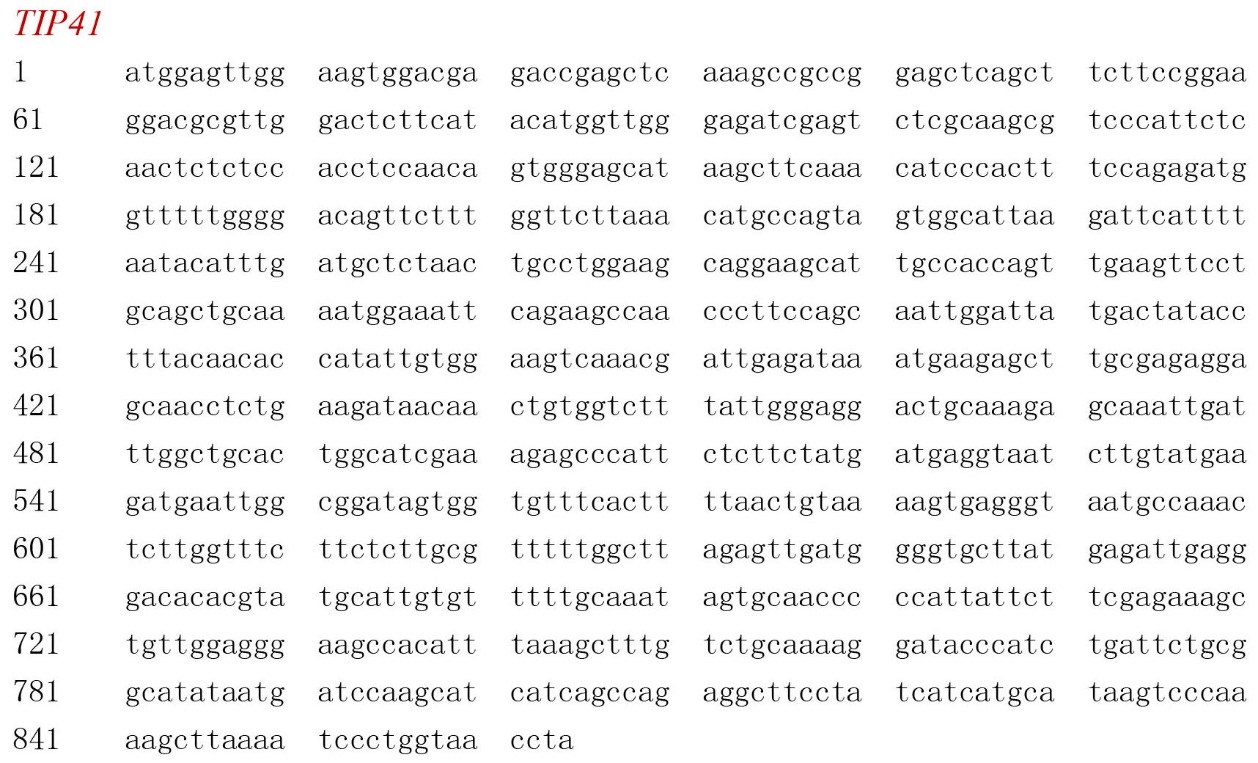


**Figure K. Nucleotide acid sequence of *TIP41* gene from *C. sinensis*.**


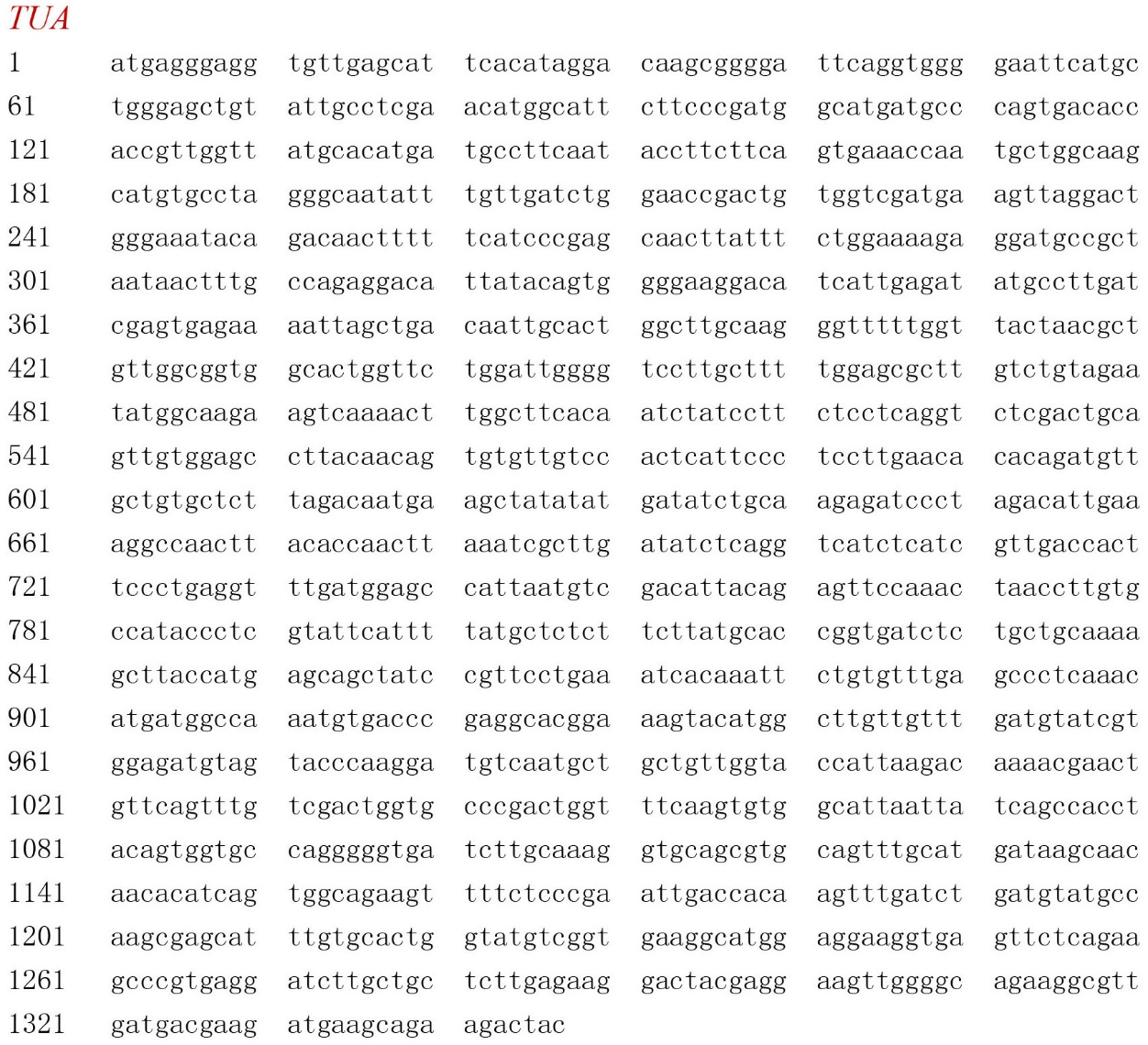


**Figure L. Nucleotide acid sequence of *TUA* gene from *C. sinensis*.**


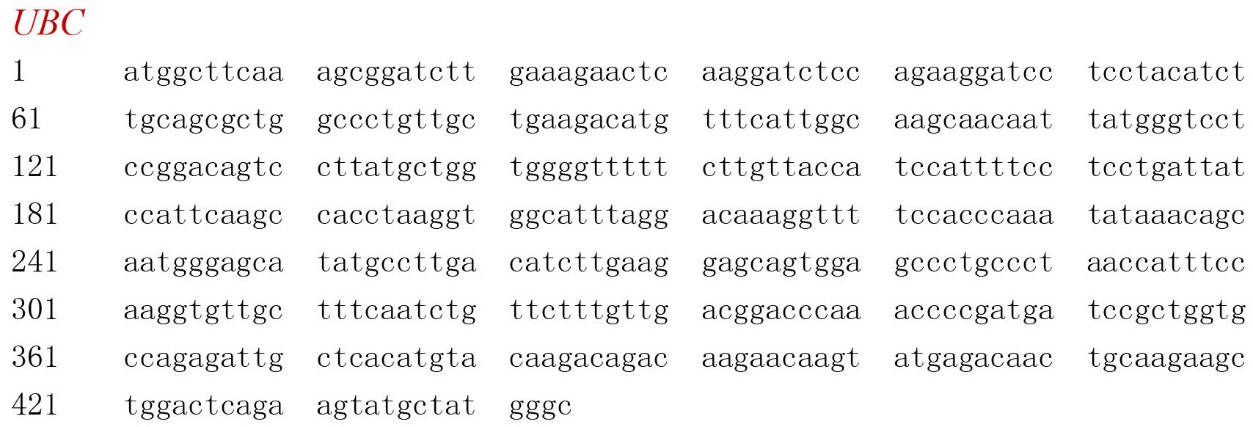


**Figure M. Nucleotide acid sequence of *UBC* gene from *C. sinensis*.**

**
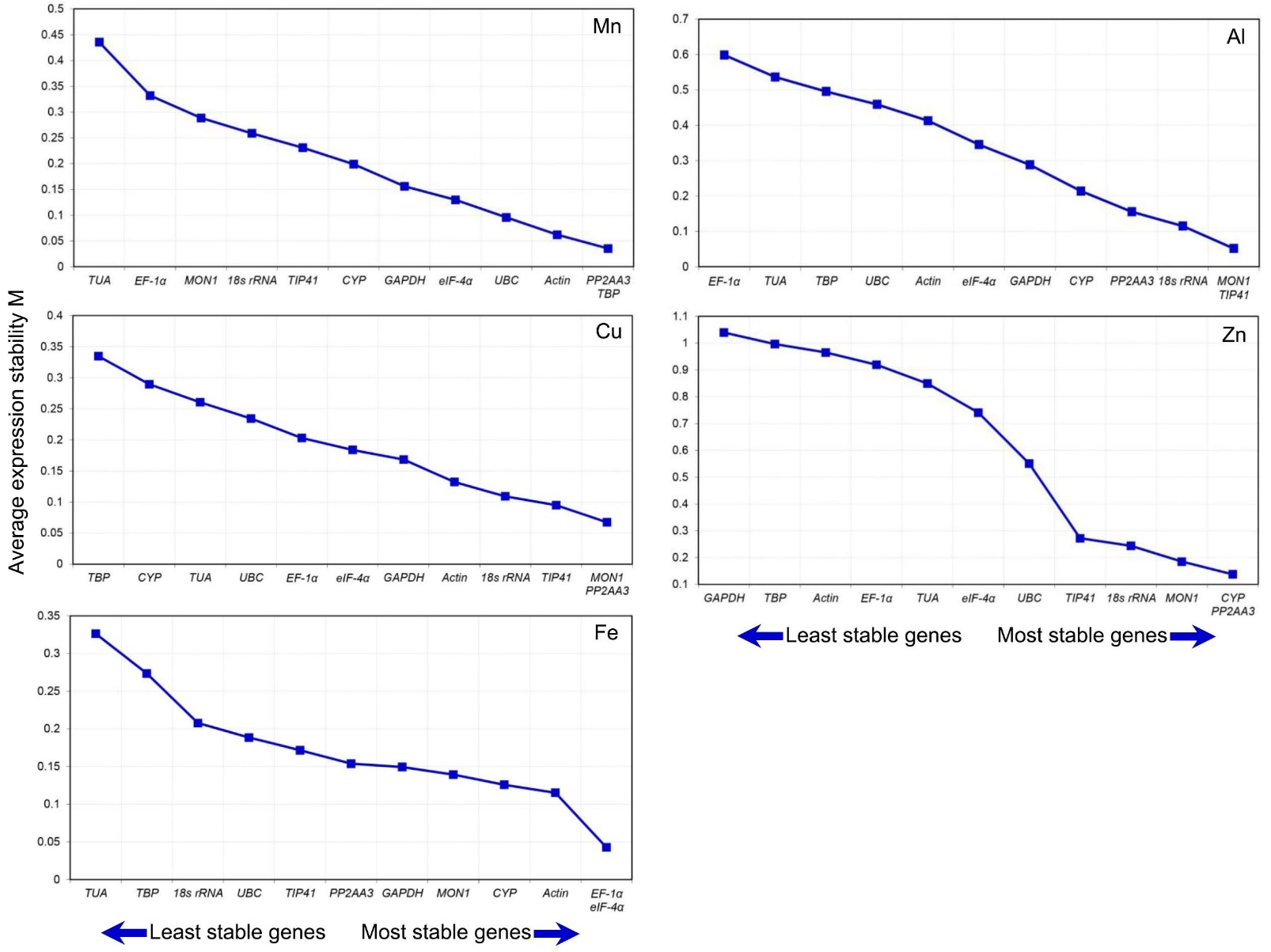
**

**Figure N. Gene expression stability values (M) and the ranking of 12 reference genes based on geNorm analyses.** The least and most stably expressed genes are on the left and right sides, respectively. Mn: MnSO_4_-treated leaves; Al: Al_2_(SO_4_)_3_-treated leaves; Cu: CuSO_4_-treated leaves; Fe: FeSO_4_-treated leaves; Zn: ZnSO_4_-treated leaves.

**Table A. Raw Cq values of the candidate reference genes in this study.** Tea plants were subjected to Mn, Al, Cu, Fe, and Zn stress treatments for 0, 1, 4, and 7 d, respectively. The samples at each time point have three biological replicates, which are indicated by 1, 2, and 3, respectively.

| **Sample name** | ***18S rRNA*** | ***Actin*** | ***EF-1α*** | ***eIF-4α*** | ***GAPDH*** | ***TUA*** | ***UBC*** | ***TBP*** | ***CYP*** | ***MON1*** | ***PP2AA3*** | ***TIP41*** |
| --- | --- | --- | --- | --- | --- | --- | --- | --- | --- | --- | --- | --- |
| Mn-0 d-1 | 18.85 | 18.57 | 18.74 | 18.53 | 18.94 | 21.73 | 17.09 | 21.74 | 23.30 | 24.12 | 23.60 | 22.69 |
| Mn-0 d-2 | 18.93 | 18.58 | 18.77 | 18.26 | 18.44 | 23.11 | 17.53 | 21.97 | 22.86 | 24.01 | 23.52 | 22.42 |
| Mn-0 d-3 | 18.66 | 18.70 | 18.48 | 17.97 | 18.07 | 22.25 | 17.24 | 22.04 | 22.88 | 23.99 | 23.34 | 22.52 |
| Mn-1 d-1 | 18.38 | 18.18 | 17.50 | 18.28 | 19.12 | 22.90 | 17.35 | 22.10 | 22.69 | 23.36 | 23.80 | 22.08 |
| Mn-1 d-2 | 18.08 | 19.13 | 18.30 | 18.43 | 18.84 | 23.20 | 17.70 | 21.91 | 22.74 | 23.49 | 23.54 | 21.95 |
| Mn-1 d-3 | 18.22 | 18.65 | 17.61 | 18.34 | 19.05 | 21.74 | 17.58 | 22.09 | 23.02 | 23.28 | 23.50 | 22.18 |
| Mn-4 d-1 | 17.97 | 18.42 | 18.65 | 18.44 | 17.57 | 20.76 | 17.06 | 22.09 | 22.40 | 24.13 | 23.15 | 22.23 |
| Mn-4 d-2 | 17.67 | 18.45 | 18.85 | 17.77 | 19.10 | 19.98 | 17.07 | 21.64 | 22.31 | 23.96 | 23.16 | 22.41 |
| Mn-4 d-3 | 18.00 | 18.32 | 18.83 | 18.03 | 18.26 | 20.66 | 17.59 | 21.37 | 22.38 | 23.71 | 23.31 | 22.23 |
| Mn-7 d-1 | 17.90 | 17.78 | 18.19 | 18.18 | 18.18 | 20.94 | 17.38 | 21.12 | 22.09 | 23.56 | 23.18 | 22.13 |
| Mn-7 d-2 | 26.13 | 18.66 | 17.82 | 18.16 | 18.45 | 20.89 | 17.43 | 21.32 | 21.84 | 23.33 | 23.13 | 22.26 |
| Mn-7 d-3 | 18.56 | 18.60 | 19.05 | 18.67 | 18.29 | 21.00 | 16.96 | 22.21 | 22.42 | 23.48 | 23.08 | 22.32 |
| Al-0 d-1 | 18.03 | 17.13 | 16.20 | 16.77 | 17.69 | 18.00 | 15.80 | 21.86 | 22.71 | 23.57 | 23.22 | 22.32 |
| Al-0 d-2 | 17.99 | 17.15 | 15.49 | 18.01 | 16.29 | 19.43 | 14.49 | 21.35 | 22.46 | 23.52 | 23.27 | 22.24 |
| Al-0 d-3 | 17.73 | 16.70 | 15.41 | 17.37 | 18.06 | 20.31 | 15.51 | 21.69 | 22.51 | 23.45 | 23.19 | 22.23 |
| Al-1 d-1 | 17.65 | 16.63 | 17.42 | 16.95 | 17.69 | 20.53 | 14.99 | 21.39 | 22.62 | 23.01 | 23.12 | 21.69 |
| Al-1 d-2 | 17.61 | 16.61 | 17.30 | 16.99 | 17.51 | 18.51 | 14.99 | 20.60 | 22.50 | 22.92 | 23.09 | 21.59 |
| Al-1 d-3 | 17.64 | 16.86 | 17.17 | 18.18 | 16.67 | 20.94 | 15.67 | 20.51 | 22.52 | 22.84 | 22.87 | 21.85 |
| Al-4 d-1 | 18.12 | 17.64 | 15.56 | 19.27 | 18.53 | 19.14 | 15.69 | 20.36 | 22.42 | 23.52 | 23.22 | 22.51 |
| Al-4 d-2 | 18.09 | 18.17 | 17.19 | 17.99 | 18.52 | 19.07 | 14.69 | 22.69 | 22.30 | 23.84 | 23.15 | 22.32 |
| Al-4 d-3 | 17.98 | 17.31 | 17.47 | 18.11 | 18.45 | 17.98 | 15.48 | 21.72 | 22.47 | 23.84 | 23.28 | 22.40 |
| Al-7 d-1 | 18.00 | 18.17 | 16.03 | 17.28 | 17.44 | 18.70 | 16.63 | 21.37 | 22.32 | 23.47 | 23.04 | 22.14 |
| Al-7 d-2 | 17.83 | 18.05 | 16.53 | 17.28 | 17.27 | 19.47 | 16.10 | 19.75 | 22.52 | 23.42 | 23.11 | 22.29 |
| Al-7 d-3 | 17.91 | 17.92 | 15.77 | 16.94 | 17.53 | 20.27 | 16.06 | 23.66 | 22.10 | 23.39 | 23.21 | 22.21 |
| Cu-0 d-1 | 18.11 | 18.37 | 18.38 | 18.37 | 18.60 | 21.48 | 16.97 | 24.13 | 22.74 | 23.48 | 23.39 | 22.31 |
| Cu-0 d-2 | 18.05 | 18.77 | 17.93 | 18.43 | 18.36 | 21.25 | 16.67 | 23.04 | 22.63 | 23.53 | 23.12 | 22.33 |
| Cu-0 d-3 | 17.88 | 18.32 | 18.26 | 18.11 | 18.27 | 20.88 | 16.90 | 23.55 | 22.53 | 23.24 | 23.13 | 22.24 |
| Cu-1 d-1 | 18.38 | 19.14 | 18.18 | 18.94 | 18.92 | 21.93 | 17.49 | 25.02 | 23.03 | 23.59 | 23.37 | 22.46 |
| Cu-1 d-2 | 18.09 | 18.54 | 18.25 | 18.37 | 18.80 | 21.75 | 17.56 | 24.14 | 22.97 | 23.53 | 23.32 | 22.27 |
| Cu-1 d-3 | 18.16 | 18.83 | 17.99 | 18.86 | 18.95 | 22.59 | 17.28 | 25.28 | 22.62 | 23.37 | 23.27 | 22.33 |
| Cu-4 d-1 | 18.03 | 18.39 | 17.67 | 18.63 | 18.95 | 22.17 | 17.42 | 24.28 | 22.23 | 23.37 | 23.27 | 22.10 |
| Cu-4 d-2 | 18.09 | 18.87 | 17.86 | 18.97 | 18.83 | 21.27 | 17.54 | 25.25 | 22.19 | 23.41 | 22.89 | 22.59 |
| Cu-4 d-3 | 18.44 | 18.92 | 18.10 | 18.96 | 18.92 | 20.45 | 17.32 | 24.26 | 21.96 | 23.33 | 22.97 | 22.09 |
| Cu-7 d-1 | 18.07 | 18.69 | 18.64 | 18.89 | 19.03 | 21.06 | 18.29 | 25.36 | 22.36 | 23.60 | 23.19 | 22.85 |
| Cu-7 d-2 | 18.22 | 18.29 | 18.83 | 19.10 | 19.00 | 23.00 | 17.41 | 25.23 | 22.31 | 23.40 | 23.44 | 22.48 |
| Cu-7 d-3 | 18.87 | 19.03 | 18.17 | 19.10 | 19.36 | 21.78 | 18.29 | 25.11 | 22.38 | 23.54 | 23.30 | 22.44 |
| Fe-0 d-1 | 18.90 | 19.10 | 18.53 | 18.34 | 18.53 | 22.31 | 16.86 | 21.28 | 23.67 | 24.39 | 24.01 | 23.23 |
| Fe-0 d-2 | 18.96 | 18.69 | 18.65 | 18.39 | 18.72 | 22.11 | 17.03 | 21.26 | 23.48 | 24.34 | 24.02 | 23.16 |
| Fe-0 d-3 | 18.78 | 18.47 | 17.19 | 18.41 | 18.61 | 22.21 | 16.83 | 21.29 | 23.48 | 24.21 | 23.96 | 23.07 |
| Fe-1 d-1 | 19.25 | 18.71 | 17.55 | 18.27 | 18.84 | 21.28 | 17.12 | 19.29 | 23.03 | 24.04 | 23.96 | 22.74 |
| Fe-1 d-2 | 19.15 | 18.20 | 17.75 | 18.13 | 18.69 | 21.41 | 17.01 | 20.06 | 23.14 | 23.99 | 24.01 | 22.73 |
| Fe-1 d-3 | 19.15 | 18.48 | 18.05 | 18.02 | 18.39 | 21.34 | 17.06 | 20.20 | 22.99 | 24.02 | 23.86 | 22.63 |
| Fe-4 d-1 | 18.63 | 18.58 | 18.39 | 18.46 | 18.32 | 21.42 | 17.23 | 20.04 | 23.15 | 23.96 | 23.71 | 23.03 |
| Fe-4 d-2 | 18.55 | 18.10 | 17.98 | 18.29 | 18.30 | 21.64 | 17.08 | 20.64 | 23.01 | 23.89 | 23.63 | 22.86 |
| Fe-4 d-3 | 18.79 | 18.45 | 17.71 | 18.33 | 19.03 | 21.67 | 17.23 | 20.62 | 23.12 | 23.86 | 23.81 | 22.82 |
| Fe-7 d-1 | 18.72 | 18.27 | 17.56 | 18.20 | 18.39 | 20.57 | 16.58 | 20.79 | 23.09 | 24.19 | 24.01 | 23.19 |
| Fe-7 d-2 | 18.87 | 18.48 | 18.10 | 17.99 | 18.48 | 20.67 | 17.00 | 20.91 | 23.04 | 24.02 | 23.80 | 23.16 |
| Fe-7 d-3 | 18.64 | 18.27 | 17.71 | 18.12 | 18.59 | 20.78 | 17.41 | 20.96 | 23.12 | 24.13 | 23.92 | 23.13 |
| Zn-0 d-1 | 17.42 | 18.45 | 16.75 | 19.15 | 19.17 | 18.17 | 16.53 | 26.07 | 22.00 | 22.45 | 22.03 | 21.45 |
| Zn-0 d-2 | 17.29 | 18.82 | 16.40 | 19.49 | 19.46 | 20.32 | 17.05 | 26.03 | 21.96 | 23.07 | 22.29 | 21.05 |
| Zn-0 d-3 | 17.34 | 19.25 | 16.63 | 19.38 | 19.44 | 20.89 | 15.49 | 22.28 | 21.72 | 22.71 | 22.56 | 21.07 |
| Zn-1 d-1 | 17.50 | 24.31 | 20.55 | 21.08 | 23.93 | 23.56 | 20.91 | 30.80 | 21.96 | 21.49 | 22.40 | 21.12 |
| Zn-1 d-2 | 17.37 | 21.26 | 17.10 | 23.15 | 22.24 | 23.80 | 15.68 | 29.32 | 21.38 | 22.48 | 22.40 | 21.11 |
| Zn-1 d-3 | 17.65 | 22.60 | 20.12 | 25.95 | 23.22 | 23.24 | 19.47 | 29.72 | 21.60 | 22.46 | 22.12 | 20.96 |
| Zn-4 d-1 | 17.32 | 22.36 | 19.63 | 21.94 | 23.16 | 25.52 | 19.29 | 29.53 | 22.02 | 23.23 | 22.82 | 21.42 |
| Zn-4 d-2 | 17.18 | 21.81 | 18.83 | 21.21 | 22.73 | 24.15 | 17.22 | 27.90 | 22.32 | 22.44 | 22.43 | 20.94 |
| Zn-4 d-3 | 17.39 | 21.60 | 21.47 | 22.46 | 23.06 | 20.13 | 18.88 | 29.36 | 22.07 | 22.36 | 22.47 | 21.08 |
| Zn-7 d-1 | 18.17 | 23.10 | 20.87 | 25.14 | 24.44 | 22.41 | 21.02 | 29.86 | 22.07 | 23.35 | 22.79 | 22.06 |
| Zn-7 d-2 | 18.09 | 23.56 | 20.72 | 24.08 | 23.56 | 27.46 | 15.35 | 29.74 | 22.77 | 22.43 | 22.91 | 22.26 |
| Zn-7 d-3 | 18.04 | 20.92 | 17.64 | 20.93 | 23.46 | 22.36 | 19.96 | 31.23 | 22.53 | 23.17 | 22.71 | 22.29 |

**Table B. Data statistics of Cq values of 12 candidate reference genes in this study.** Each Cq value is the mean value of three biological replicates from raw Cq values. SD: standard deviation.

|  | ***18S rRNA*** | ***Actin*** | ***CYP*** | ***EF-1α*** | ***eIF-4α*** | ***GAPDH*** | ***MON1*** | ***PP2AA3*** | ***TBP*** | ***TIP41*** | ***TUA*** | ***UBC*** |
| --- | --- | --- | --- | --- | --- | --- | --- | --- | --- | --- | --- | --- |
| Mn-0 d | 18.81 | 18.62 | 23.01 | 18.66 | 18.25 | 18.48 | 24.04 | 23.49 | 21.92 | 22.54 | 22.36 | 17.29 |
| Mn-1 d | 18.23 | 18.65 | 22.82 | 17.80 | 18.35 | 19.00 | 23.38 | 23.61 | 22.03 | 22.07 | 22.61 | 17.54 |
| Mn-4 d | 17.88 | 18.40 | 22.36 | 18.78 | 18.08 | 18.31 | 23.93 | 23.21 | 21.70 | 22.29 | 20.47 | 17.24 |
| Mn-7 d | 18.20 | 18.35 | 22.12 | 18.35 | 18.34 | 18.31 | 23.46 | 23.13 | 21.55 | 22.24 | 20.94 | 17.26 |
| Al-0 d | 17.92 | 16.99 | 22.56 | 15.70 | 17.38 | 18.01 | 23.51 | 23.23 | 21.63 | 22.26 | 19.58 | 15.27 |
| Al-1 d | 17.63 | 16.70 | 22.55 | 17.30 | 17.37 | 17.29 | 22.92 | 23.03 | 20.83 | 21.71 | 19.99 | 15.22 |
| Al-4 d | 18.06 | 17.71 | 22.40 | 16.74 | 18.46 | 18.50 | 23.73 | 23.22 | 22.26 | 22.41 | 19.06 | 15.29 |
| Al-7 d | 17.91 | 18.05 | 22.31 | 16.11 | 17.17 | 17.41 | 23.43 | 23.12 | 22.59 | 22.21 | 19.81 | 16.26 |
| Cu-0 d | 18.01 | 18.49 | 22.63 | 18.19 | 18.30 | 18.41 | 23.42 | 23.21 | 23.57 | 22.29 | 21.20 | 16.85 |
| Cu-1 d | 18.21 | 18.84 | 22.87 | 18.14 | 18.72 | 18.89 | 23.50 | 23.32 | 24.81 | 22.35 | 22.09 | 17.44 |
| Cu-4 d | 18.19 | 18.73 | 22.13 | 17.88 | 18.85 | 18.90 | 23.37 | 23.04 | 24.60 | 22.26 | 21.30 | 17.43 |
| Cu-7 d | 18.39 | 18.67 | 22.35 | 18.55 | 19.03 | 19.13 | 23.51 | 23.31 | 25.23 | 22.59 | 21.95 | 18.00 |
| Fe-0 d | 18.88 | 18.75 | 23.54 | 18.12 | 18.38 | 18.62 | 24.31 | 24.00 | 21.28 | 23.15 | 22.21 | 16.91 |
| Fe-1 d | 19.18 | 18.46 | 23.05 | 17.78 | 18.14 | 18.64 | 24.02 | 23.94 | 19.85 | 22.70 | 21.34 | 17.06 |
| Fe-4 d | 18.66 | 18.38 | 23.09 | 18.03 | 18.36 | 18.55 | 23.90 | 23.72 | 20.43 | 22.90 | 21.58 | 17.18 |
| Fe-7 d | 18.74 | 18.34 | 23.08 | 17.79 | 18.10 | 18.49 | 24.11 | 23.91 | 20.89 | 23.16 | 20.67 | 17.00 |
| Zn-0 d | 17.35 | 18.84 | 21.89 | 16.59 | 19.34 | 19.36 | 22.74 | 22.29 | 26.13 | 21.19 | 20.46 | 16.36 |
| Zn-1 d | 17.51 | 22.06 | 21.65 | 19.26 | 22.39 | 23.13 | 22.14 | 22.31 | 29.95 | 21.06 | 23.53 | 18.69 |
| Zn-4 d | 17.30 | 21.92 | 22.14 | 19.98 | 21.87 | 22.98 | 22.68 | 22.57 | 29.26 | 21.15 | 23.27 | 18.46 |
| Zn-7 d | 18.10 | 22.53 | 22.46 | 19.74 | 21.72 | 23.82 | 22.98 | 22.80 | 29.61 | 22.20 | 22.41 | 18.78 |
| Median | 18.14 | 18.55 | 22.50 | 18.08 | 18.36 | 18.59 | 23.48 | 23.22 | 22.15 | 22.28 | 21.32 | 17.21 |
| Mean | 18.16 | 18.87 | 22.55 | 17.97 | 18.83 | 19.21 | 23.45 | 23.22 | 23.51 | 22.24 | 21.34 | 17.08 |
| Minimum | 17.30 | 16.70 | 21.65 | 15.70 | 17.17 | 17.29 | 22.14 | 22.29 | 19.85 | 21.06 | 19.06 | 15.22 |
| Maximum | 19.18 | 22.53 | 23.54 | 19.98 | 22.39 | 23.82 | 24.31 | 24.00 | 29.95 | 23.16 | 23.53 | 18.78 |
| SD | 0.50 | 1.49 | 0.46 | 1.08 | 1.43 | 1.79 | 0.53 | 0.47 | 3.03 | 0.57 | 1.20 | 1.00 |

**Table C. Primers used for clone of the 12 candidate reference genes from *C. sinensis*.**

| Gene symbol | Primer sequence (5ʹ–3ʹ) forward/reverse | Amplicon length (bp) |
| --- | --- | --- |
| *18S rRNA* | ATGAAGTTGGAATCGCTAGTAATC/  CGACTTCACCCCAGTCGAAGACCC | 216 |
| *Actin* | ATGGCTGATGCTGAGGATATTCAG/  AAAGCACTTCCTGTGGACAATGGA | 1,131 |
| *CYP* | ATGTCGAACCCAAAGGTTTTCTTT/  ATTCTCGGTTATCTGACCACAATC | 522 |
| *EF-1α* | ATGGGTAAGGAAAAAGTTCACATT/  TTTCTTCTTCAGAGCAGCCTTGGT | 1,341 |
| *eIF-4α* | ATGGCAGGTTTGGCACCCGAAGCT/  GAGGAGATCGGCAACATTTGATGG | 1,239 |
| *GAPDH* | ATGGGCAAGATCAAGATCGGAATC/  GCAAGTGTTTGCCATGTGAACGAT | 1,011 |
| *MON1* | ATGGGGTCCGATTTGGACTCCTCA/  CCATGAAAAGGGGCTGGCTCCCAA | 1,887 |
| *PP2AA3* | ATGTCAATGGGTGATGAGCCACTG/  GCTAGACATCATGACATGATCAAT | 1,761 |
| *TBP* | ATGGCGGATCAAGTGTTGGAAGG/  CCATTGTTGATTCTTCCTGAACTC | 603 |
| *TIP41* | ATGGAGTTGGAAGTGGACGAGACC/  TAGGTTACCAGGGATTTTAAGC | 864 |
| *TUA* | ATGAGGGAGGTGTTGAGCATTCAC/  GTAGTCTTCTGCTTCATCTTCGTC | 1,347 |
| *UBC* | ATGGCTTCAAAGCGGATCTTGA/  GCCCATAGCATACTTCTGAGTCC | 444 |

**Table D. Gene expression stability ranked by NormFinder software.** Mn: MnSO_4_-treated leaves; Al: Al_2_(SO_4_)_3_-treated leaves; Cu: CuSO_4_-treated leaves; Fe: FeSO_4_-treated leaves; Zn: ZnSO_4_-treated leaves.

| **Rank** | **Mn** | |  | **Al** | |  | **Cu** | |  | **Fe** | |  | **Zn** | |
| --- | --- | --- | --- | --- | --- | --- | --- | --- | --- | --- | --- | --- | --- | --- |
|  | **Gene name** | **Stability** |  | **Gene name** | **Stability** |  | **Gene name** | **Stability** |  | **Gene name** | **Stability** |  | **Gene name** | **Stability** |
| 1 | *Actin* | 0.019 |  | *18S rRNA* | 0.030 |  | *18S rRNA* | 0.026 |  | *Actin* | 0.030 |  | *UBC* | 0.138 |
| 2 | *PP2AA3* | 0.045 |  | *PP2AA3* | 0.038 |  | *TIP41* | 0.066 |  | *eIF-4α* | 0.050 |  | *eIF-4α* | 0.178 |
| 3 | *TBP* | 0.050 |  | *TIP41* | 0.077 |  | *Actin* | 0.081 |  | *EF-1α* | 0.052 |  | *EF-1α* | 0.178 |
| 4 | *UBC* | 0.093 |  | *MON1* | 0.096 |  | *GAPDH* | 0.082 |  | *CYP* | 0.052 |  | *Actin* | 0.189 |
| 5 | *eIF-4α* | 0.106 |  | *CYP* | 0.162 |  | *MON1* | 0.084 |  | *GAPDH* | 0.064 |  | *TUA* | 0.196 |
| 6 | *CYP* | 0.116 |  | *Actin* | 0.209 |  | *PP2AA3* | 0.096 |  | *MON1* | 0.065 |  | *TBP* | 0.204 |
| 7 | *TIP41* | 0.128 |  | *GAPDH* | 0.224 |  | *eIF-4α* | 0.103 |  | *PP2AA3* | 0.080 |  | *PP2AA3* | 0.205 |
| 8 | *18S rRNA* | 0.129 |  | *eIF-4α* | 0.232 |  | *UBC* | 0.134 |  | *TIP41* | 0.116 |  | *GAPDH* | 0.211 |
| 9 | *GAPDH* | 0.149 |  | *UBC* | 0.240 |  | *TUA* | 0.140 |  | *UBC* | 0.148 |  | *18S rRNA* | 0.248 |
| 10 | *MON1* | 0.202 |  | *TBP* | 0.245 |  | *EF-1α* | 0.141 |  | *18S rRNA* | 0.165 |  | *CYP* | 0.276 |
| 11 | *EF-1α* | 0.284 |  | *TUA* | 0.313 |  | *TBP* | 0.218 |  | *TUA* | 0.242 |  | *TIP41* | 0.300 |
| 12 | *TUA* | 0.307 |  | *EF-1α* | 0.355 |  | *CYP* | 0.221 |  | *TBP* | 0.251 |  | *MON1* | 0.366 |

| **Rank** |  | **Mn** | | |  | **Al** | | |  | **Cu** | | |  | **Fe** | | |  | **Zn** | | |
| --- | --- | --- | --- | --- | --- | --- | --- | --- | --- | --- | --- | --- | --- | --- | --- | --- | --- | --- | --- | --- |
|  |  | **Gene**  **name** | **SD** | **CV** |  | **Gene**  **name** | **SD** | **CV** |  | **Gene**  **name** | **SD** | **CV** |  | **Gene**  **name** | **SD** | **CV** |  | **Gene**  **name** | **SD** | **CV** |
| 1 |  | *TIP41* | 0.16 | 0.70 |  | *PP2AA3* | 0.08 | 0.37 |  | *MON1* | 0.10 | 0.41 |  | *PP2AA3* | 0.11 | 0.45 |  | *PP2AA3* | 0.22 | 0.98 |
| 2 |  | *PP2AA3* | 0.19 | 0.83 |  | *CYP* | 0.11 | 0.50 |  | *PP2AA3* | 0.13 | 0.58 |  | *MON1* | 0.14 | 0.57 |  | *CYP* | 0.27 | 1.21 |
| 3 |  | *eIF-4α* | 0.19 | 1.06 |  | *18S rRNA* | 0.16 | 0.88 |  | *TIP41* | 0.16 | 0.71 |  | *eIF-4α* | 0.13 | 0.70 |  | *18S rRNA* | 0.28 | 1.61 |
| 4 |  | *UBC* | 0.21 | 1.19 |  | *TIP41* | 0.22 | 1.00 |  | *18S rRNA* | 0.19 | 1.02 |  | *CYP* | 0.17 | 0.75 |  | *MON1* | 0.39 | 1.73 |
| 5 |  | *MON1* | 0.29 | 1.20 |  | *MON1* | 0.24 | 1.02 |  | *GAPDH* | 0.22 | 1.15 |  | *TIP41* | 0.19 | 0.81 |  | *TIP41* | 0.41 | 1.93 |
| 6 |  | *Actin* | 0.23 | 1.23 |  | *GAPDH* | 0.47 | 2.65 |  | *EF-1α* | 0.24 | 1.30 |  | *UBC* | 0.15 | 0.89 |  | *eIF-4α* | 1.09 | 5.10 |
| 7 |  | *TBP* | 0.30 | 1.38 |  | *Actin* | 0.52 | 3.02 |  | *Actin* | 0.25 | 1.33 |  | *GAPDH* | 0.17 | 0.93 |  | *TUA* | 1.37 | 6.12 |
| 8 |  | *CYP* | 0.34 | 1.49 |  | *UBC* | 0.48 | 3.12 |  | *eIF-4α* | 0.29 | 1.54 |  | *18S rRNA* | 0.18 | 0.96 |  | *Actin* | 1.33 | 6.24 |
| 9 |  | *18S rRNA* | 0.33 | 1.81 |  | *TBP* | 0.70 | 3.19 |  | *UBC* | 0.34 | 1.94 |  | *Actin* | 0.19 | 1.03 |  | *EF-1α* | 1.67 | 8.83 |
| 10 |  | *GAPDH* | 0.40 | 2.18 |  | *eIF-4α* | 0.60 | 3.40 |  | *TUA* | 0.57 | 2.63 |  | *EF-1α* | 0.35 | 1.97 |  | *UBC* | 1.85 | 10.24 |
| 11 |  | *EF-1α* | 0.44 | 2.46 |  | *TUA* | 0.83 | 3.52 |  | *CYP* | 0.64 | 2.85 |  | *TUA* | 0.39 | 2.22 |  | *GAPDH* | 1.92 | 10.38 |
| 12 |  | *TUA* | 0.47 | 2.92 |  | *EF-1α* | 0.91 | 3.88 |  | *TBP* | 0.71 | 3.08 |  | *TBP* | 0.44 | 2.54 |  | *TBP* | 2.14 | 12.47 |

**Table E. Stability analysis of reference genes assayed by BestKeeper software.** Mn: MnSO_4_-treated leaves; Al: Al_2_(SO_4_)_3_-treated leaves; Cu: CuSO_4_-treated leaves; Fe: FeSO_4_-treated leaves; Zn: ZnSO_4_-treated leaves. SD: standard deviation; CV: coefficient of variation.
